# EMA approved orphan medicines since the implementation of the orphan legislation

**DOI:** 10.1186/s13023-025-03756-7

**Published:** 2025-06-02

**Authors:** Eveliina Hahl, Terhi Kurko, Hanna Koskinen, Marja Airaksinen, Kati Sarnola

**Affiliations:** 1 Faculty of Pharmacy, University of Helsinki, Helsinki, Finland; 2 Research Unit, The Social Insurance Institution of Finland, Helsinki, Finland

**Keywords:** Rare disease, Orphan medicine, Selection of medicines, Orphan medicines legislation, Europe

## Abstract

**Background:**

In the European Union (EU), the orphan legislation, aiming to increase the number of pharmacotherapies available for rare diseases, came into force in April 2000. This study examined the development of the selection of orphan medicines granted marketing authorisation, their approved indications, and the number of orphan medicines developed for paediatric use in EU during 2000–2022. This study also examined the availability of the orphan medicines with a marketing authorisation in the Finnish market in order to demonstrate their country level uptake in a single member state.

**Methods:**

The material on orphan medicines’ marketing authorisations and their introduction were collected from the European Commission’s Community Registers in June 2022 and analysed with a qualitative document analysis. This study covered the period 2000–2022 since the introduction of the orphan legislation, and comparisons were made in 10-year periods of, 2001–2010 and 2011–2020.

**Results:**

By May 2022, there were 213 novel orphan medicines approved in Europe during the observation period. Of them, 67% (n = 142) were on the market in Finland in May 2024. The number of new orphan medicines approved in Europe doubled from 63 products in 2001–2010 to 127 products in 2011–2020. Several orphan medicines were developed for certain type of rare diseases, such as haematological cancers. The proportion of orphan medicines approved for paediatric use decreased from 55% in 2001–2010 to 42% in 2011–2020.

**Conclusion:**

The number of orphan medicines available within EU increased significantly after the orphan legislation came into force. The development of orphan medicines seemed to often focus on diseases or disease groups that already have available treatment options, while several rare diseases remain without available treatment. Even though rare diseases are more common in children, orphan medicines have not been developed for paediatric use in the same proportion.

**Supplementary Information:**

The online version contains supplementary material available at 10.1186/s13023-025-03756-7.

## Introduction

In the European Union (EU), the orphan legislation, i.e., Regulation (EC) No 141/2000 of the European Parliament and of the Council, came into force in April 2000 [1]. The European Commission grants the orphan designation and the marketing authorisations of the orphan medicines (referred as The European Medicines Agency (EMA) approved orphan medicines). Orphan medicines are medicinal products for the diagnosis, prevention, and treatment of rare diseases [2]. The purpose of the orphan legislation is to stimulate research and development of medicinal products for the treatment of rare diseases [3].

Since the implementation of the orphan legislation, it has been possible to apply for an orphan designation for a promising medicinal product in its developmental stage [3]. Orphan designation provides access to scientific advice on ways to generate evidence on a medicine’s quality, benefits, and risks, reductions in administrative fees, and market exclusivity after marketing authorisation. Furthermore, in orphan medicine development, marketing authorisation can often be obtained after smaller-scale trials without treatment comparison, randomisation, and blinding, as the prevalence and number of patients with rare diseases is low [4].

Orphan designation is not the same as marketing authorisation and, thus, does not permit the large-scale clinical use of the medicine [5]. Orphan designation is often applied for in the early stages of medicine development, while marketing authorisation is applied for once there is sufficient evidence on the effects and risks of the medicine. Conditional marketing authorisation and marketing authorisation approved under exceptional circumstances can be used as a fast-track authorisation of orphan medicines. In such cases, marketing authorisation is granted before submitting comprehensive data on the efficacy and safety of the medicine [6]. Incentives for the research, development, and market approval of orphan medicines strive to ensure that patients suffering from rare diseases have access to the same quality of treatment than other patients [3]. 

Rare diseases refer to life threatening or chronically debilitating conditions affecting not more than 5 in 10,000 people [2]. Rare diseases is a heterogeneous group of chronic and progressive diseases that can affect any organ system, typically including various symptoms caused by, for example, degenerative or proliferative changes in the human body [6–8]. Some examples of rare diseases are cystic fibrosis, spinal muscular atrophy, patent ductus arteriosus (PDA), renal-cell carcinoma, glioma and acute myeloid leukaemia [6]. Rare diseases affect approximately 4%–6% of the world’s population, equalling to 263–446 million people worldwide and 18–30 million people in the EU [9]. Approximately 70% of rare diseases occur only in children. More than 6,000 rare diseases have been identified, and together they may form as much as one tenth of all human diseases [6, 9, 10]. No effective treatment methods are available for many rare diseases, increasing concern and suffering among patients and their families [7, 8, 11].

Even though the implementation of orphan legislation increased the number of orphan medicines developed for the treatment of rare diseases and approved for market, the legislation has also raised concern and criticism [12, 13]. The incentives of the orphan legislation are enticing, while the requirements of the traditional marketing authorisation process are heavy, leading some pharmaceutical companies to focus excessively on the development of orphan medicines [13]. The advantages brought about by the orphan legislation should be precluded without smothering the interest to develop medicines for the treatment of rare disease [12].

EMA publishes annual reports with statistics on orphan medicinal products (onwards orphan medicines) [4]. However, to the best of our knowledge, no research has been conducted on the development of the selection of the orphan medicines in EU since the implementation of the orphan legislation. The aim of this study was to examine the development of the selection of orphan medicines and their therapeutic indications as well as the number of orphan medicines developed for paediatric use in EU since the orphan legislation came into force, i.e., the period of 2000–2022. Furthemore, this study examined the availability of the orphan medicine with a marketing authorisation in the Finnish market in order to demonstrate their country level uptake in a single EU member state. We expect that the uptake in a single member state provides some overall understanding of the country level uptake of these medicines.

## Materials and methods

The research material (Supplementary material 1) was collected from the European Commission’s Community Register of orphan medicinal products and European Commission’s Community Register of not active orphan medicinal products in June 2022 [14, 15]. The European Commission’s registers of medicinal products include the implementing decisions on marketing authorisations for orphan medicines in Europe, updates and extensions of marketing authorisations, and summaries of product characteristics. Furthemore, research material (Supplementary material 2) has been collected from Finland, from the National Insurance Institution Kela’s Medicinal Products Database in May 2024. The database includes the medicinal products marketed in Finland and their reimbursement details. In this study, the applied inclusion criteria were orphan designation and marketing authorisation granted between January 2000 and May 2022. The group of medicines fulfilling these criteria is later referred as a selection of orphan medicines. Medicines with orphan designation but no marketing authorisation granted during the aforementioned period were excluded from the research material. Thus, the data comprises orphan medicinal products with orphan designation and marketing authorisation at the end of May 2022, products with valid marketing authorisation for orphan medicines and expired orphan designation (= not active orphan medicines), and products that were granted marketing authorisation for orphan medicines but whose marketing authorisation had later been cancelled.

We used qualitative document analysis, where we classified and quantified data on orphan medicines. Descriptive methods (means, percentages) and Microsoft Excel (16.70) were used in the analysis of the research material. The following data were examined more closely: the annual number of new orphan medicines and extensions of therapeutic indications, the number of conditional marketing authorisations and marketing authorisations approved under exceptional circumstances, the number of active substances, the number of different therapeutic indications, and the number of orphan medicines on the market in Finland. In addition, therapeutic indications were classified into inborn errors of metabolism or immune disorders, cancer, and other rare diseases based on their indications, described in a study by Onakpoya et al. (2015) [16]. Furthermore, we studied the number of orphan medicines with paediatric or paediatric and adult indication or subsequent paediatric extension of indication and compared it with the number of orphan medicines approved only for adults. We chose Finland as an example of the country level uptake of the medicines because Finland is relatively high-income country with high level, tax payed health care system. Therefore, we expect that orphan medicines are quite often brought in the market here.

Orphan medicines with orphan designation and valid marketing authorisation in May 2022 and orphan medicines with expired orphan designation are specified in the research material, and all products are included in the analysis. In this study we examined the entire period since the introduction of the orphan legislation, i.e., 2000–2022 and compared the following 10-year periods: 2001–2010 and 2011–2020. In the classification based on the paediatric regulation (EC) No 1901/2006, medicines intended for paediatric use are medicines approved for children aged 17 or younger, and medicines intended for adult use are medicines approved for individuals aged 18 or older. In this study, orphan medicines with approved paediatric or paediatric and adult indication at the time of marketing authorisation, orphan medicines with approved adult indication at the time of marketing authorisation and a subsequent paediatric extension of indication, and orphan medicines approved only for adults are classified separately. As the study is based on public documents from authorities, ethical review was not required.

## Results

**Fig. 1:**
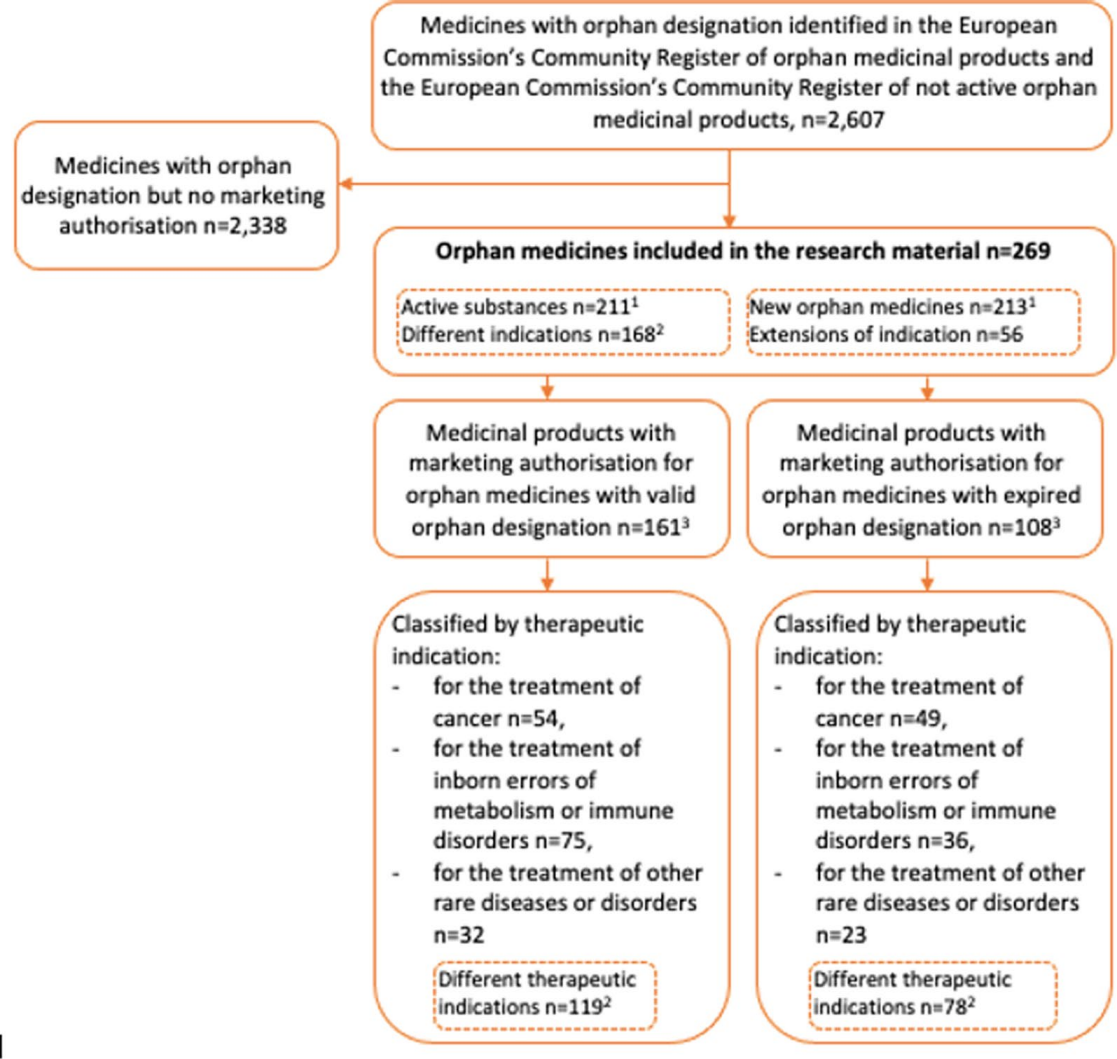
Orphan medicinal products approved in the EU during 2000–2022 and included in the research material (n = 269).^1^The number of active substances is different from the number of novel orphan medicines because two active substances were granted marketing authorisation as two new orphan medicinal products for different indications. ^2^ Same therapeutic indications may be approved for the orphan medicines in this box; therefore, this illustrates the number of completely different therapeutic indications. ^3^Including extensions of indications

We identified 269 orphan medicines for 168 different indications that had been granted marketing authorisation since the introduction of the EU orphan legislation in 2000 (Fig. 1, Additional file 1). Of them, 79% (n = 213) were novel orphan medicines. The remaining 21% (n = 56) were extensions of existing marketing authorisations to cover new therapeutic indications. Orphan medicines with marketing authorisations included 211 active substances (differing from the number of novel orphan medicines, n = 213). Two active substances, everolimus and cholic acid, were granted marketing authorisation as two new orphan medicinal products for different indications.

**Fig. 2:**
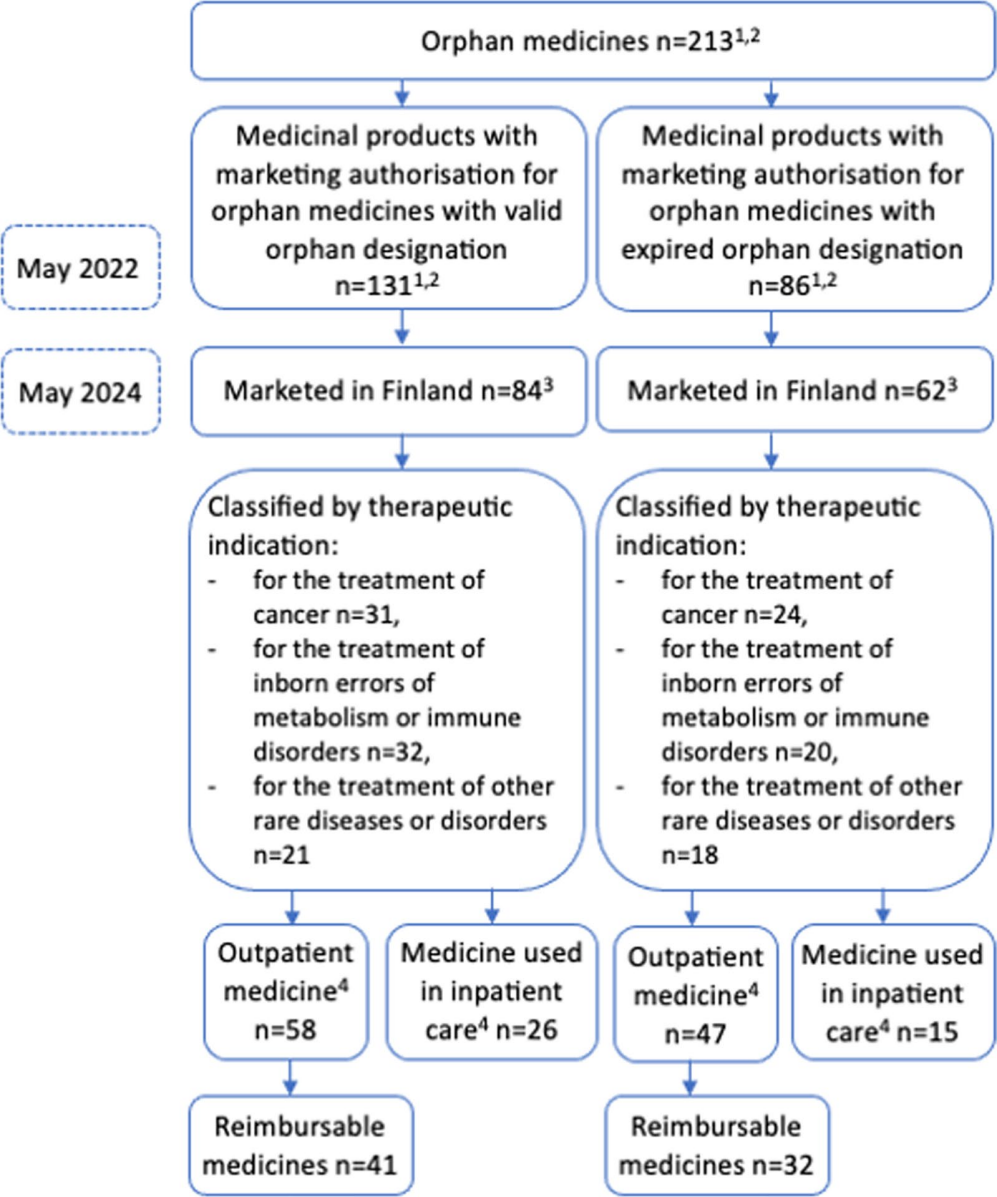
Orphan medicinal products on the market in Finland in May 2024 (n = 142). ^1^Not including extensions of indications. ^2^The number of orphan medicines is different from the sum of orphan medicines with valid orphan designation and orphan medicine with expired orphan designation because four medicinal products have valid and expired orphan designation for different indications. ^3^Four medicinal products had valid and expired orphan designation for different indications. ^4^Classified by dosage form. Infusions are classified as medicine used in inpatient care; other medicines are classified as outpatient medicines

Of the 213 novel orphan medicines being approved in EU by May 2022, two-thirds (67%, n = 142) were on the market in Finland in May 2024 (Fig. 2, Supplementary material 2). Of the orphan medicines on the market in Finland, 38% (n = 54) were indicated for the treatment of cancer, and 35% (n = 50) were indicated for the treatment of inborn errors of metabolism or immune system disorders. Furthermore, 27% (n = 38) of the orphan medicines were indicated for the treatment of other rare diseases.

### The selection of orphan medicines

**Fig. 3:**
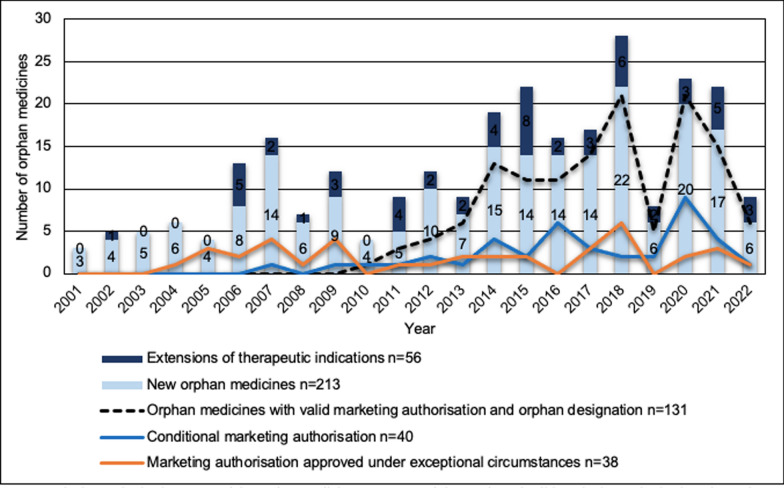
Marketing authorisations granted for orphan medicines per year and the number of valid marketing authorisations for orphan-designated medicines on 31 May 2022

The selection of orphan medicines has developed and the annual number of new marketing authorisations has increased within EU since the beginning of the 2000 s, when the European orphan legislation was implemented (Fig. 3). The number of novel orphan medicines with a valid marketing authorisation and orphan designation was 131 in May 2022. Between 2001 and 2010, marketing authorisations were granted to 63 new orphan medicines, averaging 6.3 orphan medicines per year.

During the following 10-year period, i.e., 2011–2020, the corresponding number was 127, averaging 12.7 new orphan medicines per year. A noticeable peak in the number of marketing authorisations granted to new orphan medicines was detected in 2018 (n = 22). In addition to this, extensions of therapeutic indications were authorised for six new orphan medicines that year. During the following year, marketing authorisations were granted to only six new orphan medicines, and extensions of therapeutic indications were authorised for two orphan medicines. Since 2014, the number of marketing authorisations for new orphan medicines has remained at more than 10 per year, and there has been an upwards trend during the reference period.

A 10-year period of market exclusivity, with an additional 2 years for medicines that have complied with an agreed Paediatric Investigation Plan (PIP), is included in the incentives of the orphan legislation. Therefore, at the end of May 2022, marketing authorisations were valid for orphan-designated medicinal products that were granted marketing authorisation in 2010 or later (Fig. 3). Between 2000 and 2021, the number of conditional marketing authorisations and the number of marketing authorisations approved under exceptional circumstances per year were 1.9 and 1.8, respectively. Since 2007, the trend of orphan medicines approved with a conditional marketing authorisation has been slightly increasing. In 2020, the number of conditional marketing authorisations was the largest, 10, which equals to more than 50% of all marketing authorisations granted for new orphan medicines that year. Relative to the number of marketing authorisations granted in one year, the most marketing authorisations under exceptional circumstances were granted in 2005, i.e., 75% (n = 3). For the most part, the share of marketing authorisations approved under exceptional circumstances has remained at the same low level, suggesting that these types of marketing authorisations are not pre-eminently utilised when bringing new orphan medicines into the market.

### Indications for orphan medicines

**Fig. 4:**
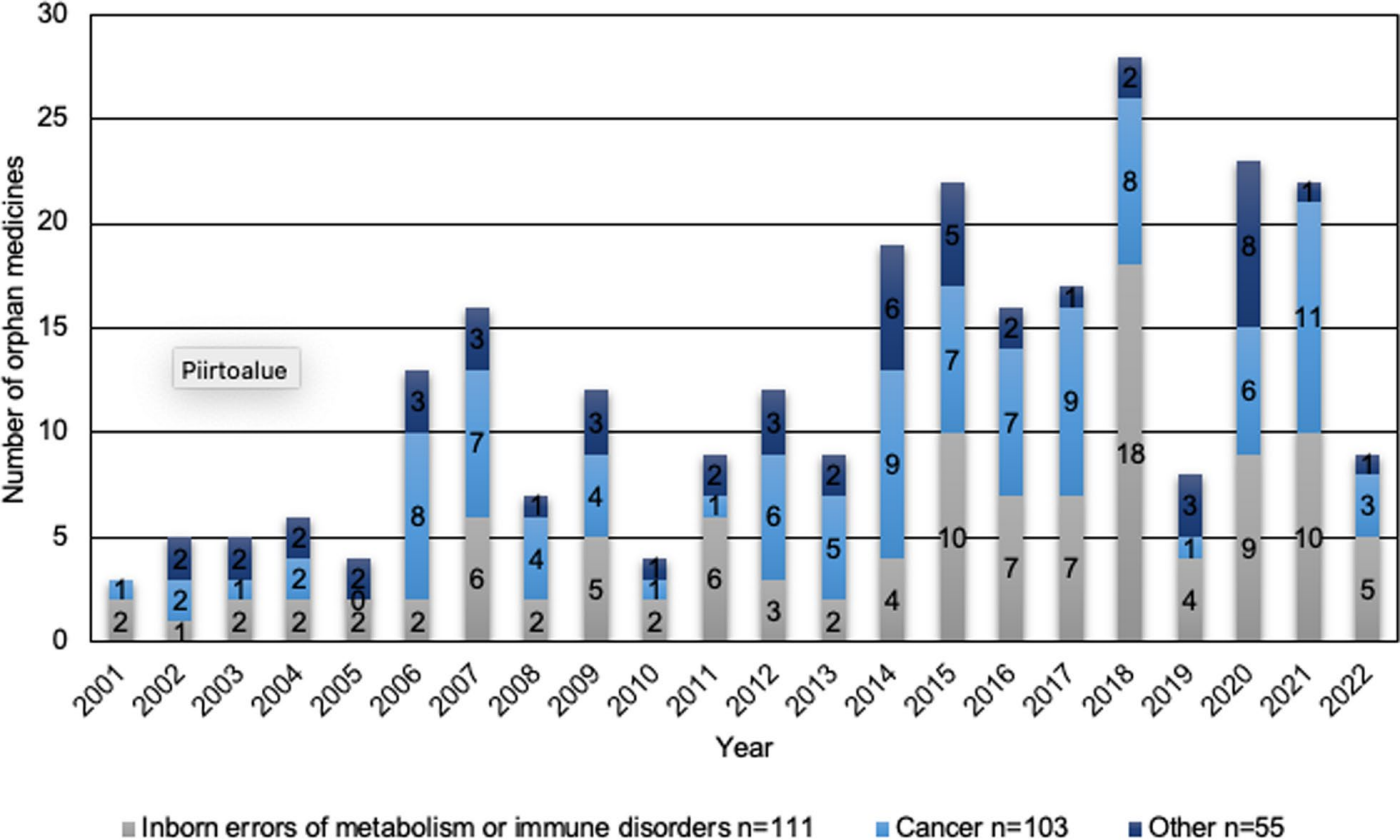
Orphan medicines classified by marketing authorisations per year and divided into three categories according to therapeutic indications: inborn errors of metabolism or immune disorders; cancer; and other diseases

When classified by therapeutic indications, 41% (n = 111) of orphan medicines with marketing authorisations granted during the study period were indicated for the treatment of inborn errors of metabolism or immune system disorders, and 38% (n = 103) were indicated for the treatment of cancer (Fig. 4). Furthermore, 20% (n = 55) of orphan medicines were granted marketing authorisation for the treatment of other rare diseases.

During the first 10 years after the orphan legislation came into force (2001–2010), 35% (n = 26) of marketing authorisations for orphan medicines were granted to medicines for inborn errors of metabolism or immune disorders, while the corresponding proportion for the subsequent 10-year period, i.e., 2011–2020, was 43% (n = 70). Of all marketing authorisations for orphan medicines, 40% (n = 30) and 36% (n = 59) were granted for the treatment of cancer in 2001–2010 and 2011–2020, respectively. Correspondingly, 25% (n = 19) of orphan medicines in 2001–2010 and 21% (n = 34) in 2011–2020 were granted marketing authorisation for other rare diseases. Based on the number of orphan medicines with marketing authorisation, the focus in the development of orphan indications appears to have shifted slightly from medicines developed for the treatment of cancer to medicines developed for the treatment of inborn errors of metabolism or immune disorders.

Table 1 presents all rare diseases with three or more approved orphan medicines as well as the number of orphan medicines approved for the treatment of said disease. The number of orphan medicines developed for treating the same diseases was the largest in orphan medicines developed for the treatment of cancer (Table 1). Blood cancers were the most common indications for orphan medicines, and most orphan medicines were developed for the treatment of acute myeloid leukaemia (n = 8) and multiple myeloma (n = 8). Other leukaemias (n = 3) and various lymphomas (n = 3) were also among the indications for orphan medicines developed for the treatment of the most common types of cancer.

**Table 1:** Indications (n = 24) with three or more orphan medicines with marketing authorisation, and number of medicines in a class with granted marketing authorisation

Classified by therapeutic indication	Indication	Number of orphan medicines
Inborn errors of metabolism or immune disorders	Treatment of mucopolysaccharidosis	5
	Treatment of cystic fibrosis	4
	Treatment of Gaucher disease	3
	Treatment of Fabry disease	3
	Treatment of sickle cell disease	3
	Treatment of primary bile acid synthesis	3
	Treatment of growth hormone deficiency	3
	Treatment of ATTR amyloidosis	3
The total number of orphan medicines with marketing authorisation in class		111
Cancer	Treatment of acute myeloid leukaemia	8
	Treatment of multiple myeloma	8
	Treatment of acute lymphoblastic leukaemia	7
	Treatment of chronic myeloid leukaemia	5
	Treatment of diffuse large B- cell lymphoma	4
	Treatment of myelodysplastic syndromes	4
	Treatment of ovarian cancer	4
	Treatment of chronic lymphocytic leukaemia	4
	Treatment of mantle cell lymphoma	4
	Treatment of renal cell carcinoma	3
	Treatment of cutaneous T-cell lymphoma	3
The total number of orphan medicines with marketing authorisation in class		103
Other	Infections (other than tuberculosis)	8
	Treatment of pulmonary arterial hypertension	7
	Treatment of severe epilepsies in childhood	5
	Treatment of tuberculosis	4
	Conditioning regimen for haematopoietic cell transplantation	3
The total number of orphan medicines with marketing authorisation in class		55

Orphan medicines developed for the treatment of inborn errors of metabolism or immune disorders were most commonly indicated for the treatment of mucopolysaccharidoses (n = 5). However, each of the orphan medicines approved for the treatment of mucopolysaccharidoses were approved for the treatment of a different type of mucopolysaccharidosis. Cystic fibrosis was the second most common indication (n = 4). Three orphan medicines were developed for the treatment of the other inborn errors of metabolism or immune disorders included in Table 1.

Orphan medicines developed for the treatment of other rare diseases were most commonly indicated for various infections (n = 8). Each orphan medicine developed for the treatment of an infection was approved for the treatment of a different infection, and the indications included viral, bacterial, and fungal infections. Of the infections, tuberculosis was listed separately, as four orphan medicines were developed for the treatment of the disease. In this class, other common indications included pulmonary hypertension (n = 7), severe epilepsies in childhood (n = 5), such as Dravet syndrome or Lennox-Gastaut syndrome, and conditioning regimen for haematopoietic cell transplantation (n = 3).

### Orphan medicines approved for paediatric use

**Fig. 5:**
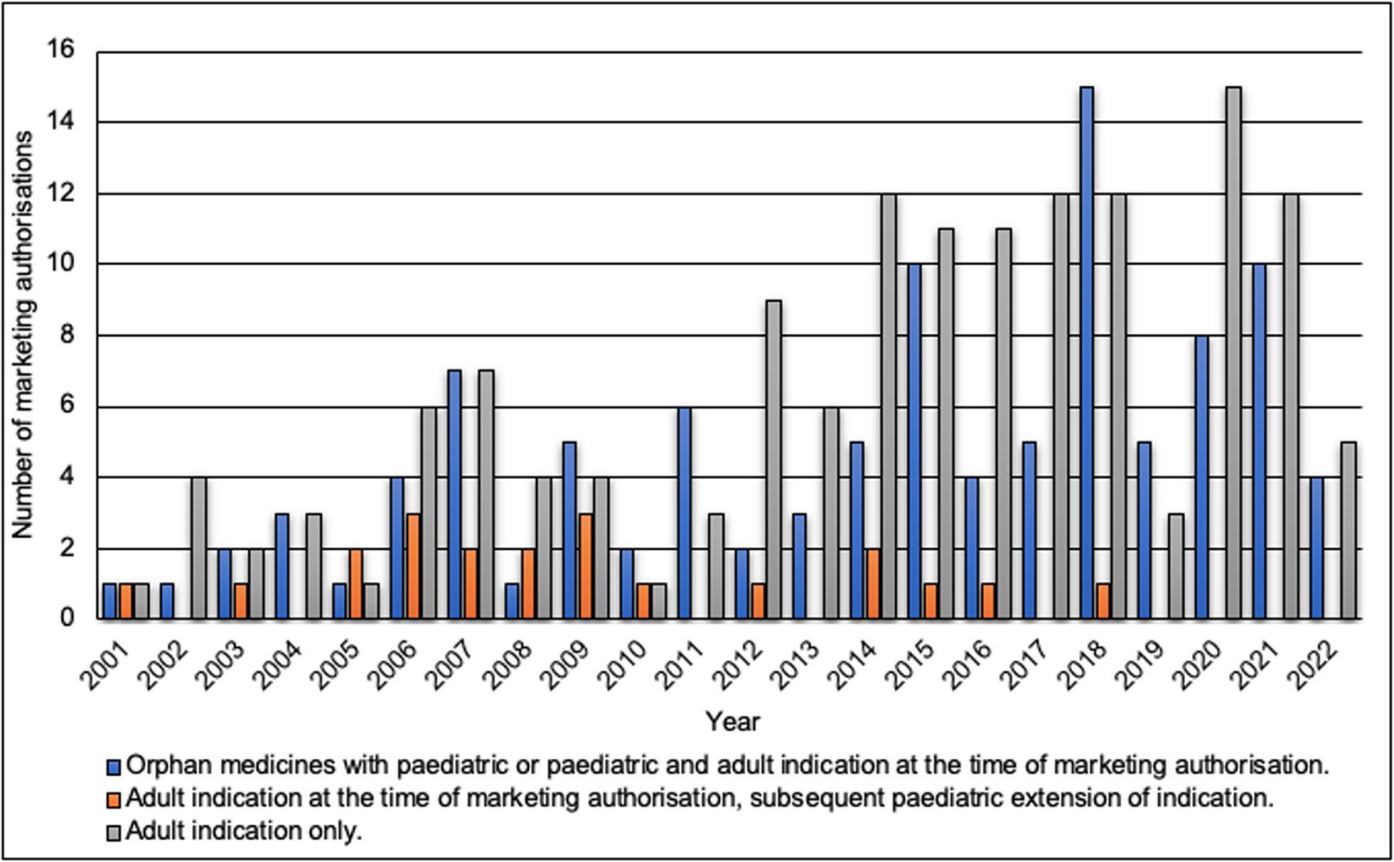
Orphan medicines approved for paediatric or paediatric and adult indication (n = 124) compared to orphan medicines approved only for adults (n = 145)

Since the orphan legislation came into force, the share of orphan medicines approved for paediatric or paediatric and adult use has been 39% (n = 104) of all orphan medicines with marketing authorisation (Fig. 5). If subsequent paediatric extensions of indications are included, the share is 46% (n = 124). Between 2001 and 2010, the first 10 years after the implementation of the orphan legislation, the share of orphan medicines approved for paediatric use, including paediatric extensions of indications, was 55% (n = 41) of all orphan medicines with marketing authorisation. During the following 10 years, i.e., 2011–2020, the share dropped to 42% (n = 69). Paediatric extensions of indications were approved almost every year until 2010 (n = 15) (Fig. 5), after which they were only approved in 2012, 2014–2016, and 2018 (n = 6).

In relation to all marketing authorisations granted to orphan medicines in a year, the largest share were granted to orphan medicines approved for paediatric or paediatric and adult use, paediatric extensions included, in 2005 (75%) and 2010 (75%). In 7 years between 2001 and 2010, more than 50% of orphan medicines with marketing authorisation were approved for paediatric or paediatric and adult use either straight away or through paediatric extension of indication. Between 2011 and 2020, there were only 4 years when at least half of all approved medicines were approved for paediatric or paediatric and adult use. The proportional share of orphan medicines approved for paediatric use has decreased in the past 10 years (2011–2020), even though the number of orphan medicines approved for paediatric use surpassed the corresponding number from 2001–2010 by 28.

## Discussion

In this study, we examined the development in the number of orphan medicines, therapeutic indications, and orphan medicines approved for paediatric use after the implementation of the EU orphan legislation. This study, along with reports form the European Medicines Agency [17], show that the number of orphan medicines with marketing authorisation has increased significantly in EU since the introduction of the orphan legislation in 2000. During the first 10-year period, i.e., 2001–2010, 63 new orphan medicinal products were approved. The number of new orphan medicines doubled to 127 over the next 10 years. Since the orphan legislation came into force, there has been a continuously growing trend in marketing authorisations granted for orphan medicines**.** The number of new orphan medicines has increased rather steadily every year, but an observable peak was detected in 2018. Since 2007, the trend of orphan medicines approved with a conditional marketing authorisation has been slightly increasing. Conditional marketing authorisation can be used to fast-track the approval of medicines that addresses unmet medical needs of patients.

The increasing number of orphan medicines brings hope of new treatment options to those with rare diseases who are in the most vulnerable positions. However, the increasing number of orphan medicines does not automatically mean the new treatments are effective, safe, and available to all patients with rare diseases [16, 18–20]. This study also sheds light to national level implementation of orphan therapies using Finland as a case. We expect that the uptake in a single member state provides some overall understanding of the country level uptake of the orphan medicines, although further research is needed to compare uptake in other high-income countries.

Since the implementation of the orphan legislation in 2000, the focus in the development of orphan medicines has slightly shifted from medicines developed for the treatment of cancer to medicines developed for the treatment of inborn errors of metabolism and immune disorders. However, the differences in the number of medicines approved are small: The number of orphan medicines approved for the treatment of inborn errors of metabolism or immune system disorders surpasses the number of orphan medicines approved for the treatment of cancer by eight during the nearly 22-year period after the introduction of the orphan legislation. Blood cancers were the most common indication for orphan medicines, which could be explained by the generally active development of cancer medicines in the 2000 s [21].

Because of the incentives of the orphan legislation, pharmaceutical companies have become increasingly more interested in developing medicines for rare diseases [12, 13]. However, medicine development does not necessarily target the rarest rare diseases with no available treatment. Instead, pharmaceutical companies may be striving to take advantage of the orphan legislation and save on the costs of medicine development and market access while developing medicines for the most common rare diseases or pursuing wider markets through, for example, extensions of indications [12, 13, 18]. There are several medicines available for the treatment of many rare diseases, and medicine development appears to focus on specific diseases and disease groups. The results of this study support this argument, as orphan designation and marketing authorisation have been granted to several medicinal products for the treatment of the same diseases.

According to the EU orphan legislation, there should be no satisfactory method of treatment with marketing authorisation, or new orphan medicines should be of significant benefit when compared to existing treatment of diseases treated with orphan medicines [2]. Yet, the results of this study show that four or more orphan medicines have been approved for the treatment of several cancers and seven orphan medicines have been approved for the treatment of pulmonary hypertension. However, additional research is needed to study the basis for granting these medicines an orphan designation, whether all orphan medicines approved for the treatment of the same rare diseases are on the market, and what the significant benefit of these medicines is compared to other available treatment options. On the other hand, a majority of the 6,000 identified rare diseases remain without available treatment [9, 18]. The orphan legislation should be developed to match its original purpose so that medicine development would also target diseases with no available treatment.

Several of the rare diseases appear at birth or in childhood, including spinal muscular atrophy, lysosomal storage disorders, familial adenomatous polyposis (FAP) and cystic fibrosis [6]. In the 22 years since the introduction of the orphan legislation, only 46% of orphan medicines with marketing authorisation have been approved for paediatric or paediatric and adult use, even though 70% of rare diseases affect only children [9]. The low number of medicines developed for paediatric use has been noted earlier as well [22]. Furthermore, the relative share of orphan medicines with marketing authorisation approved for paediatric or paediatric and adult use was 55% of all orphan medicines approved for sale between 2001 and 2010. During the following 10 years, i.e., 2011–2020, the corresponding number was 42%. The current trend in orphan medicines does not correspond to the prevalence of rare diseases in children. In addition to the EU orphan legislation, also other regulatory reforms, such as the Paediatric legislation and the Paediatric Investigation Plans (PIPs), may influence the trends of medicine development for children. For instance, these regulatory reforms might have had a slowing effect on getting the paediatric indications in rare diseases.

The aim of this study was to gain an overall understanding of the development of the selection of orphan medicines for a period of over 20 years since the orphan legislation was enacted. There are many, not so well-known factors that may have influenced the development trends of orphan medicines, such as investments in medical research on rare diseases, advances in science and technology to facilitate understanding of disease etiology needed for effective treatment development, better advocacy and engagement of the patient populations, or commercial incentives. Furthermore, the growing trend of personalised medicines and advanced therapy medicinal products may influence the development trends. These underlying factors affecting the trends should be studied in the future to gain an in-depth understanding on the trends of medicine development for rare conditions.

A medicine should be effective and safe, regardless of the rarity of the disease. The orphan legislation and the lack of robust evidence regarding the therapeutic benefits of orphan medicines have been discussed recently [23]. The orphan legislation allows for smaller-scale and lower-quality trials compared to those related to non-rare diseases, which often leads to a low quantity of evidence on the efficacy and safety of the orphan medicine [4, 16]. Nevertheless, pharmaceutical companies tend to set the price of orphan medicines high, forcing society to consider the cost-effectiveness of the treatment as well as making the medicine available to patients [12, 13, 19]. Based on the findings of this study, new orphan medicines should increasingly be developed mainly for diseases with no available treatments. Furthermore, additional research should be conducted to investigate whether the orphan legislation, introduced more than 20 years ago, still meets the needs set for it. It would be important to study how the sales and life cycle of products with orphan designation have developed in the long run, and whether medicines with orphan designation have become block busters.

The strength of this study is the comprehensive research material covering the entire period since the introduction of the orphan legislation, i.e., 2000–2022. In addition, the research material is based on reliable documentation by authorities, and the material was analysed by a group of researchers. However, it should be noted that off-label use of orphan medicines occurs, i.e., medicines are used for purposes other than their original approved indication, and off-label use cannot be detected from the European Commission’s register data. Thus, this study examined the official indications of orphan medicines based on market authorisation documentation. While assessing the country-level uptake of the medicines it should be noted that we used one country as an example to gain a general understanding of the uptake. While assessing further the use of orphan medicines, it is important to remember that the prevalence of rare diseases vary between countries influencing the results. Therefore, it would be valuable to conduct comparative studies between countries to gain better understanding of the country level uptake of the orphan medicines and factors influencing it.

## Conclusions

The number of orphan medicines in EU has increased significantly since the introduction of the orphan legislation, i.e., from 2000 to 2022. The selection of orphan medicines tends to focus on medicines used for treating cancer and inborn errors of metabolism or immune system disorders. The coverage of orphan medicines is still minor compared to number of orphan diseases and it seems that the orphan medicines market is focused on rare conditions with highest prevalence. The development of orphan medicines for paediatric use has not been proportionate to the prevalence of rare diseases in children. Orphan medicines with marketing authorisation often target diseases or disease groups that already have available treatments, while several rare diseases remain without available treatment.

## Supplementary Information

Additional file 1: The research material. Table S1. Medicinal product with marketing authorisation for orphan medicines with valid orphan designation in May 2022. Table S2. Medicinal products with marketing authorisation for orphan medicines with expired orphan designation in May 2022.

Additional file 2: The research material. Table S3. Orphan medicinal products on the market in Finland in May 2024 which had valid orphan designation in May 2022. Table S4. Orphan medicinal products on the market in Finland in May 2024 which had expired orphan designation in May 2022.

## Data Availability

The authors declare that the data supporting the findings of this study are available within the paper and its Supplementary Information files. Should any raw data files be needed in another format they are available from the corresponding author upon reasonable request. Source data are provided with this paper.
