# Supplementary material for: EMA approved orphan medicines since the implementation of the orphan legislation
Source: Orphanet J Rare Dis. 2025 Jun 2;20:266. doi: 10.1186/s13023-025-03756-7 (PMC12131831; doi:10.1186/s13023-025-03756-7)
Supplement: Supplementary file 1 — Additional file 1: The research material. Table S1. Medicinal product with marketing authorisation for orphan medicines with valid orphan designation in May 2022. Table S2. Medicinal products with marketing authorisation for orphan medicines with expired orphan designation in May 2022. [file 13023_2025_3756_MOESM1_ESM.pdf]

Supplemental Information: Additional file 1 – The research material

Orphanet Journal of Rare Diseases

EMA approved orphan medicines since the implementation of the orphan legislation, Orphanet Journal of Rare Diseases

Eveliina Hahl [1,*], Terhi Kurko [2], Hanna Koskinen [2], Marja Airaksinen [1] and Kati Sarnola [2]

* Correspondence: eveliina.hahl@helsinki.fi
[1] Faculty of Pharmacy, University of Helsinki, Helsinki, Finland
[2] Research Unit, The Social Insurance Institution of Finland, Helsinki, Finland

Contents

Table S1 Medicinal product with marketing authorisation for orphan medicines with valid orphan designation in May 2022
Table S2 Medicinal products with marketing authorisation for orphan medicines with expired orphan designation in May 2022

Table S1 Medicinal product with marketing authorisation for orphan medicines with valid orphan designation

Indication for children (adults are aged 18 years or older)

Tradename	Active substance	Indication	Classification	Designation date	Date of authorisation	Conditional marketing authorisation	Marketing authorisation witch to non-conditional	Authorisation under exceptional circumstances	New orphan medicine	May 2022	2 additional years of market exclusivity as paediatric reward	Marketing authorisation holder
Abecma	idecabtagene vicleucel	Treatment of multiple myeloma	cancers	20-Apr-2017	18-Aug-2021	x						Bristol-Myers Squibb Pharma EEIG
Adakveo	crizanlizumab	Treatment of sickle cell disease	inborn errors of metabolism or immune disorders	9-Aug-2012	28-Oct-2020	x			x	x		Novartis Europharm Limited
Adcetris	brentuximab vedotin	Treatment of cutaneous T-cell lymphoma	cancers	11-Jan-2012	15-Dec-2017	x	x (05/2022)					Takeda Pharma A/S
Adcetris	brentuximab vedotin	Treatment of peripheral T-cell lymphoma	cancers	15-Jan-2009	25-Oct-2012	x	x (05/2022)					Takeda Pharma A/S
Adcetris	brentuximab vedotin	Treatment of Hodgkin lymphoma	cancers	15-Jan-2009	25-Oct-2012	x	x (05/2022)					Takeda Pharma A/S
Adempas	riociguat	Treatment of pulmonary arterial hypertension including treatment of chronic thromboembolic pulmonary hypertension	other conditions	20-Dec-2007	27-Mar-2014							Bayer AG

Indication for children (adults are aged 18 years or older)

Tradename	Active substance	Indication	Classification	Designation date	Date of authorisation	Conditional marketing authorisation	Marketing authorisation witch to non-conditional	Authorisation under exceptional circumstances	New orphan medicine	May 2022	2 additional years of market exclusivity as paediatric reward	Marketing authorisation holder
Alofisel	darvadstrocel	Treatment of anal fistula	other conditions	8-Oct-2009	23-Mar-2018							Takeda Pharma A/S
Alprolix	eftrenonacog alfa	Treatment of haemophilia B (congenital factor IX deficiency)	inborn errors of metabolism or immune disorders	8-Jun-2007	12-May-2016				x	x	x (08/2020)	Swedish Orphan Biovitrum AB (publ)
Amglidia	glibenclamide	Treatment of neonatal diabetes	inborn errors of metabolism or immune disorders	15-Jan-2016	24-May-2018				x	x		AMMTeK
Arikayce liposomal	amikacin	Treatment of nontuberculous mycobacterial lung disease	other conditions	8-Apr-2014	27-Oct-2020							Insmed Netherlands B.V.
Artesunate Amivas	artesunate	Treatment of malaria	other conditions	28-Feb-2020	22-Nov-2021				x	x		Amivas Ireland Limited
Aspaveli	pegcetacoplan	Treatment of paroxysmal nocturnal haemoglobinuria	inborn errors of metabolism or immune disorders	22-May-2017	13-Dec-2021							Swedish Orphan Biovitrum AB (publ)
Ayvakyt	avapritinib	Treatment of gastrointestinal stromal tumours	cancers	17-Jul-2017	24-Sep-2020	x						Blueprint Medicines (Netherlands) B.V.
Ayvakyt	avapritinib	Treatment of mastocytosis	cancers	26-Oct-2018	24-Mar-2022	x						Blueprint Medicines (Netherlands) B.V.
Besponsa	inotuzumab ozogamicin	Treatment of B-cell acute lymphoblastic leukaemia	cancers	7-Jun-2013	29-Jun-2017							Pfizer Europe MA EEIG
Blenrep	belantamab mafodotin	Treatment of multiple myeloma	cancers	16-Oct-2017	25-Aug-2020	x						GlaxoSmithKline (Ireland) Limited
Blincyto	blinatumomab	Treatment of acute lymphoblastic leukaemia	cancers	24-Jul-2009	23-Nov-2015	x	x (06/2018)			x		Amgen Europe B.V.
Brineura	cerliponase alfa	Treatment of neuronal ceroid lipofuscinosis type 2	inborn errors of metabolism or immune disorders	12-Mar-2013	30-May-2017			x	x	x		BioMarin International Limited
Bylvay	odevixibat	Treatment of progressive familial intrahepatic cholestasis	inborn errors of metabolism or immune disorders	17-Jul-2012	16-Jul-2021			x	x	x		Albireo AB
Cablivi	caplacizumab	Treatment of thrombotic thrombocytopenic purpura	inborn errors of metabolism or immune disorders	30-Apr-2009	31-Aug-2018					x		Ablynx N.V.
Cerdelga	eliglustat	Treatment of Gaucher Disease	inborn errors of metabolism or immune disorders	4-Dec-2007	19-Jan-2015							Genzyme Europe B.V.
Chenodeoxycholic acid Leadiant	chenodeoxycholic acid	Treatment of inborn errors of primary bile acid synthesis	inborn errors of metabolism or immune disorders	16-Dec-2014	10-Apr-2017			x	x	x		Leadiant GmbH

									Indication for children (adults are aged 18 years or older)			
**Tradename**	**Active substance**	**Indication**	**Classification**	**Designation date**	**Date of authorisation**	**Conditional marketing authorisation**	**Marketing authorisation witch to non-conditional**	**Authorisation under exceptional circumstances**	**New orphan medicine**	**May 2022**	**2 additional years of market exclusivity as paediatric reward**	**Marketing authorisation holder**
Coagadex	human coagulation factor X	Treatment of hereditary factor X deficiency	inborn errors of metabolism or immune disorders	17-Sep-2007	16-Mar-2016				x	x	x (08/2018)	BPL Bioproducts Laboratory GmbH
Cometriq	cabozantinib	Treatment of medullary thyroid carcinoma	cancers	6-Feb-2009	21-Mar-2014	x	x (09/2021)					Ipsen Pharma
Cresemba	isavuconazole	Treatment of mucormycosis	other conditions	4-Jun-2014	15-Oct-2015							Basilea Pharmaceutica Deutschland GmbH
Cresemba	isavuconazole	Treatment of invasive aspergillosis	other conditions	4-Jul-2014	15-Oct-2015							Basilea Pharmaceutica Deutschland GmbH
Crysvita	burosumab	Treatment of X-linked hypophosphataemia	inborn errors of metabolism or immune disorders	15-Oct-2014	19-Feb-2018	x			x	x		Kyowa Kirin Holdings B.V.
Cystadrops	mercaptamine	Treatment of cystinosis	inborn errors of metabolism or immune disorders	7-Nov-2008	19-Jan-2017				x	x		Recordati Rare Diseases
Dacogen	decitabine	Treatment of acute myeloid leukaemia	cancers	8-Jun-2006	20-Sep-2012						x (03/2020)	Janssen-Cilag International NV
Darzalex	daratumumab	Treatment of plasma cell myeloma	cancers	17-Jul-2013	20-May-2016	x	x (05/2017)					Janssen-Cilag International NV
Darzalex	daratumumab	Treatment of AL amyloidosis	inborn errors of metabolism or immune disorders	25-May-2018	21-Jun-2021							Janssen-Cilag International NV
Daurismo	glasdegib	Treatment of acute myeloid leukaemia	cancers	16-Oct-2017	26-Jun-2020							Pfizer Europe MA EEIG
Defitelio	defibrotide	Treatment of hepatic veno-occlusive disease	other conditions	29-Jul-2004	18-Oct-2013			x	x	x		Gentium S.r.I.
Deltyba	delamanid	Treatment of tuberculosis	other conditions	1-Feb-2008	27-Apr-2014	x				x		Otsuka Novel Products GmbH
Dovprela	pretomanid	Treatment of tuberculosis	other conditions	29-Nov-2007	31-Jul-2020	x						Mylan IRE Healthcare Ltd
Elzonris	tagraxofusp	Treatment of blastic plasmacytoid dendritic cell neoplasm	cancers	11-Nov-2016	7-Jan-2021			x				Stemline Therapeutics B.V.
Enspryng	satralizumab	Treatment of neuromyelitis optica spectrum disorders	inborn errors of metabolism or immune disorders	27-Jun-2016	24-Jun-2021				x	x		Roche Registration GmbH
Epidyolex	cannabidiol	Treatment of Dravet syndrome	other conditions	15-Oct-2014	19-Sep-2019				x	x		GW Pharma (International) B.V
Epidyolex	cannabidiol	Treatment of Lennox-Gastaut syndrome	other conditions	20-Mar-2017	19-Sep-2019				x	x		GW Pharma (International) B.V

									Indication for children (adults are aged 18 years or older)			
**Tradename**	**Active substance**	**Indication**	**Classification**	**Designation date**	**Date of authorisation**	**Conditional marketing authorisation**	**Marketing authorisation witch to non-conditional**	**Authorisation under exceptional circumstances**	**New orphan medicine**	**May 2022**	**2 additional years of market exclusivity as paediatric reward**	**Marketing authorisation holder**
Epidyolex	cannabidiol	Treatment of tuberous sclerosis	cancers	17-Jan-2018	16-Apr-2021				x	x		GW Pharma (International) B.V
Evrysdi	risdiplam	Treatment of spinal muscular atrophy	inborn errors of metabolism or immune disorders	26-Feb-2019	26-Mar-2021				x	x		Roche Registration GmbH
Farydak	panobinostat	Treatment of multiple myeloma	cancers	8-Nov-2012	28-Aug-2015							Secura Bio Limited
Fintepla	fenfluramine	Treatment of Dravet syndrome	other conditions	18-Dec-2013	18-Dec-2020				x	x		Zogenix ROI Limited
Galafold	migalastat	Treatment of Fabry disease	inborn errors of metabolism or immune disorders	22-May-2006	26-May-2016					x		Amicus Therapeutics Europe Limited
Gazyvaro	obinutuzumab	Treatment of chronic lymphocytic leukaemia	cancers	10-Oct-2012	23-Jul-2014							Roche Registration GmbH
Gazyvaro	obinutuzumab	Treatment of follicular lymphoma	cancers	19-Jun-2015	13-Jun-2016							Roche Registration GmbH
Givlaari	givosiran	Treatment of acute hepatic porphyria	inborn errors of metabolism or immune disorders	29-Aug-2016	2-Mar-2020				x	x		Alnylam Netherlands B.V.
Granupas	para-aminosalicylic acid	Treatment of tuberculosis	other conditions	17-Dec-2010	7-Apr-2014				x	x		Eurocept International B.V.
Hepcludex	bulevirtide	Treatment of hepatitis delta virus infection	other conditions	19-Jun-2015	31-Jul-2020	x						Gilead Sciences Ireland Unlimited Company
Hetlioz	tasimelteon	Treatment of non-24-hour sleep-wake disorders in blind people with no light perception	other conditions	23-Feb-2011	3-Jul-2015							Vanda Pharmaceuticals Netherlands B.V.
Holoclar	Ex vivo expanded autologous human corneal epithelial cells containing stem cells	Treatment of corneal lesions, with associated corneal (limbal) stem cell deficiency, due to ocular burns	other conditions	7-Nov-2008	17-Feb-2015	x						Holostem Terapie Avanzate S.r.l.
Iclusig	ponatinib	Treatment of acute lymphoblastic leukaemia	cancers	2-Feb-2010	1-Jul-2013							Incyte Biosciences Distribution B.V.
Iclusig	ponatinib	Treatment of chronic myeloid leukaemia	cancers	2-Feb-2010	1-Jul-2013							Incyte Biosciences Distribution B.V.
Idefirix	imlifidase	Prevention of graft rejection following solid organ transplantation	other conditions	12-Jan-2017	25-Aug-2020	x						Hansa Biopharma AB
Idelvion	albutrepenonacog alfa	Treatment of haemophilia B	inborn errors of metabolism or immune disorders	4-Feb-2010	11-May-2016				x	x		CSL Behring GmbH

Indication for children (adults are aged 18 years or older)

Tradename	Active substance	Indication	Classification	Designation date	Date of authorisation	Conditional marketing authorisation	Marketing authorisation witch to non-conditional	Authorisation under exceptional circumstances	New orphan medicine	May 2022	2 additional years of market exclusivity as paediatric reward	Marketing authorisation holder
Imcivree	setmelanotide	Treatment of pro-opiomelanocortin deficiency	inborn errors of metabolism or immune disorders	14-Jul-2016	16-Jul-2021				x	x		Rhythm Pharmaceuticals Netherlands B.V.
Imcivree	setmelanotide	Treatment of leptin receptor deficiency	inborn errors of metabolism or immune disorders	19-Nov-2018	16-Jul-2021				x	x		Rhythm Pharmaceuticals Netherlands B.V.
Imnovid (Pomalidomide Celgene)	pomalidomide	Treatment of multiple myeloma	cancers	8-Oct-2009	5-Aug-2013							Bristol-Myers Squibb Pharma EEIG
Inrebic	fedratinib	Treatment of primary myelofibrosis	cancers	1-Oct-2010	8-Feb-2021							Bristol-Myers Squibb Pharma EEIG
Inrebic	fedratinib	Treatment of post-essential thrombocythaemia myelofibrosis	cancers	26-Nov-2010	8-Feb-2021							Bristol-Myers Squibb Pharma EEIG
Inrebic	fedratinib	Treatment of post-polycythaemia vera myelofibrosis	cancers	26-Nov-2010	8-Feb-2021							Bristol-Myers Squibb Pharma EEIG
Isturisa	osilodrostat	Treatment of Cushing’s syndrome	other conditions	15-Oct-2014	9-Jan-2020							Recordati Rare Diseases
Jorveza	budesonide	Treatment of eosinophilic oesophagitis	inborn errors of metabolism or immune disorders	5-Aug-2013	8-Jan-2018							Dr Falk Pharma GmbH
Kaftrio	ivacaftor / tezacaftor / elexacaftor	Treatment of cystic fibrosis	inborn errors of metabolism or immune disorders	14-Dec-2018	21-Aug-2020				x	x		Vertex Pharmaceuticals (Ireland) Limited
Kalydeco	ivacaftor	Treatment of cystic fibrosis	inborn errors of metabolism or immune disorders	8-Jul-2008	23-Jul-2012				x	x		Vertex Pharmaceuticals (Ireland) Limited
Kanuma	sebelipase alfa	Treatment of lysosomal acid lipase deficiency	inborn errors of metabolism or immune disorders	17-Dec-2010	28-Aug-2015				x	x		Alexion Europe SAS
Ketoconazole HRA	ketoconazole	Treatment of Cushing’s syndrome	other conditions	23-Apr-2012	19-Nov-2014				x	x		HRA Pharma Rare Diseases
Kimmtrak	tebentafusp	Treatment of uveal melanoma	cancers	19-Feb-2021	1-Apr-2022							Immunocore Ireland Limited
Koselugo	selumetinib	Treatment of neurofibromatosis type 1	cancers	31-Jul-2018	17-Jun-2021	x			x	x		AstraZeneca AB
Kymriah	tisagenlecleucel	Treatment of B-lymphoblastic leukaemia/lymphoma	cancers	29-Apr-2014	23-Aug-2018				x	x		Novartis Europharm Limited
Kymriah	tisagenlecleucel	Treatment of diffuse large B-cell lymphoma	cancers	14-Oct-2016	23-Aug-2018							Novartis Europharm Limited

Indication for children (adults are aged 18 years or older)

Tradename	Active substance	Indication	Classification	Designation date	Date of authorisation	Conditional marketing authorisation	Marketing authorisation witch to non-conditional	Authorisation under exceptional circumstances	New orphan medicine	May 2022	2 additional years of market exclusivity as paediatric reward	Marketing authorisation holder
Kymriah	tisagenlecleucel	Treatment of follicular lymphoma	cancers	19-Jul-2021	29-Apr-2022							Novartis Europharm Limited
Kyprolis	carfilzomib	Treatment of multiple myeloma	cancers	3-Jun-2008	19-Nov-2015							Amgen Europe B.V.
Lamzede	velmanase alfa	Treatment of α-Mannosidosis	inborn errors of metabolism or immune disorders	26-Jan-2005	23-Mar-2018			x	x	x		Chiesi Farmaceutici S.p.A.
Ledaga	chlormethine	Treatment of cutaneous T-cell lymphoma	cancers	22-Mar-2012	3-Mar-2017							Helsinn Birex Pharmaceuticals Ltd
Libmeldy	atidarsagene autotemcel	Treatment of metachromatic leukodystrophy	inborn errors of metabolism or immune disorders	13-Apr-2007	17-Dec-2020				x	x		Orchard Therapeutics (Netherlands) B.V.
Lonapegsomatropin Ascendis Pharma	lonapegsomatropin	Treatment of growth hormone deficiency	inborn errors of metabolism or immune disorders	17-Oct-2019	11-Jan-2022				x	x		Ascendis Pharma Endocrinology Division A/S
Lutathera	lutetium (177Lu) oxodotreotide	Treatment of gastro-entero-pancreatic neuroendocrine tumours	cancers	31-Jan-2008	26-Sep-2017							Advanced Accelerator Applications
Luxturna	voretigene neparvovec	Treatment of inherited retinal dystrophies (initially named treatment of Leber's congenital amaurosis)	inborn errors of metabolism or immune disorders	2-Apr-2012	22-Nov-2018				x	x		Novartis Europharm Limited
Luxturna	voretigene neparvovec	Treatment of inherited retinal dystrophies (initially named treatment of retinitis pigmentosa)	inborn errors of metabolism or immune disorders	28-Jul-2015	22-Nov-2018				x	x		Novartis Europharm Limited
Mepsevii	vestronidase alfa	Treatment of mucopolysaccharidosis type VII (Sly syndrome)	inborn errors of metabolism or immune disorders	21-Mar-2012	23-Aug-2018			x	x	x		Ultragenyx Germany GmbH
Minjuvi	tafasitamab	Treatment of diffuse large B-cell lymphoma	cancers	15-Jan-2015	26-Aug-2021	x						Incyte Biosciences Distribution B.V.
Myalepta	metreleptin	Treatment of Familial Partial Lipodystrophy	inborn errors of metabolism or immune disorders	17-Jul-2012	30-Jul-2018			x	x	x		Amryt Pharmaceuticals Designated Activity Company
Myalepta	metreleptin	Treatment of Barraquer-Simons syndrome	inborn errors of metabolism or immune disorders	17-Jul-2012	30-Jul-2018			x	x	x		Amryt Pharmaceuticals Designated Activity Company
Myalepta	metreleptin	Treatment of Lawrence syndrome	inborn errors of metabolism or immune disorders	17-Jul-2012	30-Jul-2018			x	x	x		Amryt Pharmaceuticals

Indication for children (adults are aged 18 years or older)

Tradename	Active substance	Indication	Classification	Designation date	Date of authorisation	Conditional marketing authorisation	Marketing authorisation witch to non-conditional	Authorisation under exceptional circumstances	New orphan medicine	May 2022	2 additional years of market exclusivity as paediatric reward	Marketing authorisation holder
												Designated Activity Company
Myalepta	metreleptin	Treatment of Berardinelli-Seip syndrome	inborn errors of metabolism or immune disorders	17-Jul-2012	30-Jul-2018			x	x	x		Amryt Pharmaceuticals Designated Activity Company
Mylotarg	gemtuzumab ozogamicin	Treatment of acute myeloid leukaemia	cancers	18-Oct-2000	19-Apr-2018				x	x		Pfizer Europe MA EEIG
Namuscla	mexiletine	Treatment of myotonic disorders	inborn errors of metabolism or immune disorders	19-Nov-2014	18-Dec-2018							Lupin Europe GmbH
Natpar	parathyroid hormone	Treatment of hypoparathyroidism	inborn errors of metabolism or immune disorders	18-Dec-2013	24-Apr-2017	x						Takeda Pharmaceuticals International AG Ireland Branch
Nexavar	sorafenib	Treatment of follicular thyroid cancer	cancers	13-Nov-2013	23-May-2014							Bayer AG
Nexavar	sorafenib	Treatment of papillary thyroid cancer	cancers	13-Nov-2013	23-May-2014							Bayer AG
NexoBrid	Concentrate of proteolytic enzymes enriched in bromelain	Treatment of partial deep dermal and full thickness burns	other conditions	30-Jul-2002	18-Dec-2012							MediWound Germany GmbH
Ngenla	somatrogon	Treatment of growth hormone deficiency	inborn errors of metabolism or immune disorders	24-Jan-2013	14-Feb-2022				x	x		Pfizer Europe MA EEIG
Ninlaro	ixazomib	Treatment of multiple myeloma	cancers	27-Sep-2011	21-Nov-2016	x						Takeda Pharma A/S
Obiltoxaximab SFL	obiltoxaximab	Treatment of anthrax	other conditions	24-Aug-2018	18-Nov-2020			x	x	x		SFL Pharmaceuticals Deutschland GmbH
Ocaliva	obeticholic acid	Treatment of primary biliary cirrhosis	inborn errors of metabolism or immune disorders	27-Jul-2010	12-Dec-2016	x						Intercept Pharma International Limited
Onivyde pegylated liposomal	irinotecan	Treatment of pancreatic cancer	cancers	9-Dec-2011	14-Oct-2016							Les Laboratoires Servier
Onpattro	patisiran	Treatment of transthyretin-mediated amyloidosis	inborn errors of metabolism or immune disorders	15-Apr-2011	27-Aug-2018							Alnylam Netherlands B.V.
Opsumit	macitentan	Treatment of pulmonary arterial hypertension	other conditions	27-Sep-2011	20-Dec-2013							Janssen-Cilag International NV

									Indication for children (adults are aged 18 years or older)			
**Tradename**	**Active substance**	**Indication**	**Classification**	**Designation date**	**Date of authorisation**	**Conditional marketing authorisation**	**Marketing authorisation witch to non-conditional**	**Authorisation under exceptional circumstances**	**New orphan medicine**	**May 2022**	**2 additional years of market exclusivity as paediatric reward**	**Marketing authorisation holder**
Orphacol	cholic acid	Treatment of inborn errors in primary bile acid synthesis	inborn errors of metabolism or immune disorders	18-Dec-2002	12-Sep-2013			x	x	x		Laboratoires CTRS
Oxbryta	voxelotor	Treatment of sickle cell disease	inborn errors of metabolism or immune disorders	18-Nov-2016	14-Feb-2022				x	x		Global Blood Therapeutics Netherlands B.V.
Oxervate	cenegermin	Treatment of neurotrophic keratitis	inborn errors of metabolism or immune disorders	14-Dec-2015	6-Jul-2017							Dompé farmaceutici S.p.A.
Oxlumo	lumasiran	Treatment of primary hyperoxaluria	inborn errors of metabolism or immune disorders	21-Mar-2016	19-Nov-2020				x	x		Alnylam Netherlands B.V.
Palynziq	pegvaliase	Treatment of hyperphenylalaninaemia	inborn errors of metabolism or immune disorders	28-Jan-2010	3-May-2019				x	x		BioMarin International Limited
Pemazyre	pemigatinib	Treatment of biliary tract cancer	cancers	24-Aug-2018	26-Mar-2021	x						Incyte Biosciences Distribution B.V.
Polivy	polatuzumab vedotin	Treatment of diffuse large B-cell lymphoma	cancers	16-Apr-2018	16-Jan-2020	x	x (05/2022)					Roche Registration GmbH
Poteligeo	mogamulizumab	Treatment of cutaneous T-cell lymphoma	cancers	14-Oct-2016	22-Nov-2018							Kyowa Kirin Holdings B.V.
Prevymis	letermovir	Prevention of cytomegalovirus disease in patients with impaired cell-mediated immunity deemed at risk	other conditions	15-Apr-2011	8-Jan-2018							Merck Sharp & Dohme B.V.
Procysbi	mercaptamine (cysteamine bitartrate)	Treatment of cystinosis	inborn errors of metabolism or immune disorders	20-Sep-2010	6-Sep-2013				x	x		Chiesi Farmaceutici S.p.A.
Qarziba	dinutuximab beta	Treatment of neuroblastoma	cancers	8-Nov-2012	8-May-2017			x	x	x		EUSA Pharma (Netherlands) B.V.
Qinlock	ripretinib	Treatment of gastrointestinal stromal tumours	cancers	8-Nov-2017	18-Nov-2021							Deciphera Pharmaceuticals (Netherlands) B.V.
Ravicti	glycerol phenylbutyrate	Treatment of carbamoyl-phosphate synthase-1 deficiency	inborn errors of metabolism or immune disorders	10-Jun-2010	27-Nov-2015				x	x	x (12/2018)	Immedica Pharma AB
Ravicti	glycerol phenylbutyrate	Treatment of ornithine carbamoyltransferase deficiency	inborn errors of metabolism or immune disorders	10-Jun-2010	27-Nov-2015				x	x	x (12/2018)	Immedica Pharma AB
Ravicti	glycerol phenylbutyrate	Treatment of citrullinaemia type 1	inborn errors of metabolism or immune disorders	10-Jun-2010	27-Nov-2015				x	x	x (12/2018)	Immedica Pharma AB

									Indication for children (adults are aged 18 years or older)			
**Tradename**	**Active substance**	**Indication**	**Classification**	**Designation date**	**Date of authorisation**	**Conditional marketing authorisation**	**Marketing authorisation witch to non-conditional**	**Authorisation under exceptional circumstances**	**New orphan medicine**	**May 2022**	**2 additional years of market exclusivity as paediatric reward**	**Marketing authorisation holder**
Ravicti	glycerol phenylbutyrate	Treatment of argininosuccinic aciduria	inborn errors of metabolism or immune disorders	10-Jun-2010	27-Nov-2015				x	x	x (12/2018)	Immedica Pharma AB
Ravicti	glycerol phenylbutyrate	Treatment of hyperargininaemia	inborn errors of metabolism or immune disorders	10-Jun-2010	27-Nov-2015				x	x	x (12/2018)	Immedica Pharma AB
Ravicti	glycerol phenylbutyrate	Treatment of ornithine translocase deficiency (hyperornithinaemia-hyperammonaemia homocitrullinuria (HHH) syndrome)	inborn errors of metabolism or immune disorders	10-Jun-2010	27-Nov-2015				x	x	x (12/2018)	Immedica Pharma AB
Raxone	idebenone	Treatment of Leber's hereditary optic neuropathy	inborn errors of metabolism or immune disorders	15-Feb-2007	8-Sep-2015			x	x	x		Santhera Pharmaceuticals (Deutschland) GmbH
Reblozyl	luspatercept	Treatment of beta-thalassaemia intermedia and major	inborn errors of metabolism or immune disorders	29-Jul-2014	25-Jun-2020							Bristol-Myers Squibb Pharma EEIG
Reblozyl	luspatercept	Treatment of myelodysplastic syndromes	cancers	22-Aug-2014	25-Jun-2020							Bristol-Myers Squibb Pharma EEIG
Revestive	teduglutide	Treatment of Short Bowel Syndrome	other conditions	11-Dec-2001	30-Aug-2012					x		Shire Pharmaceuticals Ireland Limited
Rydapt	midostaurin	Treatment of acute myeloid leukaemia	cancers	29-Jul-2004	18-Sep-2017							Novartis Europharm Limited
Rydapt	midostaurin	Treatment of mastocytosis	cancers	4-Aug-2010	18-Sep-2017							Novartis Europharm Limited
Scenesse	afamelanotide	Treatment of erythropoietic protoporphyria	inborn errors of metabolism or immune disorders	8-May-2008	22-Dec-2014			x				Clinuvel Europe Limited
Signifor	pasireotide	Treatment of acromegaly	other conditions	8-Oct-2009	19-Nov-2014							Recordati Rare Diseases
Sirturo	bedaquiline	Treatment of tuberculosis	other conditions	26-Aug-2005	5-Mar-2014	x				x		Janssen-Cilag International NV
Sogroya	somapacitan	Treatment of growth hormone deficiency	inborn errors of metabolism or immune disorders	24-Aug-2018	31-Mar-2021							Novo Nordisk A/S
Soliris	eculizumab	Treatment of atypical haemolytic uremic syndrome	inborn errors of metabolism or immune disorders	24-Jul-2009	24-Nov-2011				x	x	x (07/2015)	Alexion Europe SAS

									Indication for children (adults are aged 18 years or older)			
**Tradename**	**Active substance**	**Indication**	**Classification**	**Designation date**	**Date of authorisation**	**Conditional marketing authorisation**	**Marketing authorisation witch to non-conditional**	**Authorisation under exceptional circumstances**	**New orphan medicine**	**May 2022**	**2 additional years of market exclusivity as paediatric reward**	**Marketing authorisation holder**
Soliris	eculizumab	Treatment of neuromyelitis optica spectrum disorders	inborn errors of metabolism or immune disorders	5-Aug-2013	26-Aug-2019							Alexion Europe SAS
Soliris	eculizumab	Treatment of myasthenia gravis	inborn errors of metabolism or immune disorders	29-Jul-2014	14-Aug-2017							Alexion Europe SAS
SomaKit TOC	edotreotide	Diagnosis of gastro-entero-pancreatic neuroendocrine tumours	other conditions	19-Mar-2015	8-Dec-2016							Advanced Accelerator Applications
Spinraza	nusinersen	Treatment of 5q spinal muscular atrophy	inborn errors of metabolism or immune disorders	2-Apr-2012	30-May-2017				x	x	x (11/2018)	Biogen Netherlands B.V.
Strensiq	asfotase alfa	Treatment of hypophosphatasia	inborn errors of metabolism or immune disorders	3-Dec-2008	28-Aug-2015			x	x	x		Alexion Europe SAS
Strimvelis	autologous CD34+ enriched cell fraction that contains CD34+ cells transduced with retroviral vector that encodes for the human ADA cDNA sequence	Treatment of severe combined immunodeficiency (SCID) due to adenosine deaminase (ADA) deficiency	inborn errors of metabolism or immune disorders	26-Aug-2005	26-May-2016				x	x	x (05/2016)	Orchard Therapeutics (Netherlands) B.V.
Sylvant	siltuximab	Treatment of Castleman's disease	cancers	30-Nov-2007	22-May-2014							EUSA Pharma (Netherlands) B.V.
Symkevi	tezacaftor/ivacaftor	Treatment of cystic fibrosis	inborn errors of metabolism or immune disorders	27-Feb-2017	31-Oct-2018				x	x		Vertex Pharmaceuticals (Ireland) Limited
Takhzyro	lanadelumab	Treatment of hereditary angioedema	inborn errors of metabolism or immune disorders	9-Oct-2015	22-Nov-2018				x	x		Takeda Pharmaceuticals International AG Ireland Branch
Tavneos	avacopan	Treatment of microscopic polyangiitis	inborn errors of metabolism or immune disorders	19-Nov-2014	11-Jan-2022							Vifor Fresenius Medical Care Renal Pharma France
Tavneos	avacopan	Treatment of granulomatosis with polyangiitis	inborn errors of metabolism or immune disorders	19-Nov-2014	11-Jan-2022							Vifor Fresenius Medical Care Renal Pharma France
Tecartus	brexucabtagene autoleucel	Treatment of mantle cell lymphoma	cancers	13-Nov-2019	14-Dec-2020	x						Kite Pharma EU B.V.
Tegsedi	inotersen	Treatment of ATTR amyloidosis	inborn errors of metabolism or immune disorders	26-Mar-2014	6-Jul-2018							Akcea Therapeutics Ireland Limited

									Indication for children (adults are aged 18 years or older)			
**Tradename**	**Active substance**	**Indication**	**Classification**	**Designation date**	**Date of authorisation**	**Conditional marketing authorisation**	**Marketing authorisation witch to non-conditional**	**Authorisation under exceptional circumstances**	**New orphan medicine**	**May 2022**	**2 additional years of market exclusivity as paediatric reward**	**Marketing authorisation holder**
Tobi Podhaler	tobramycin	Treatment of Pseudomonas aeruginosa lung infection in cystic fibrosis	other conditionst	17-Mar-2003	20-Jul-2011				x	x	x (01/2015)	Mylan IRE Healthcare Ltd
Translarna	ataluren	Treatment of Duchenne muscular dystrophy	inborn errors of metabolism or immune disorders	27-Mar-2005	31-Jul-2014	x			x	x		PTC Therapeutics International Limited
Trecondi	treosulfan	Conditioning treatment prior to haematopoietic progenitor cell transplantation	other conditions	23-Feb-2004	20-Jun-2019				x	x		medac Gesellschaft für klinische Spezialpräparate mbH
Trepulmix	treprostinil sodium	Treatment of chronic thromboembolic pulmonary hypertension	other conditions	8-Feb-2013	3-Apr-2020							SciPharm Sàrl
Verkazia	ciclosporin	Treatment of vernal keratoconjunctivitis	inborn errors of metabolism or immune disorders	6-Apr-2006	6-Jul-2018				x	x	x (07/2018)	Santen Oy
Vimizim	elosulfase alfa	Treatment of mucopolysaccharidosis, type IVA (Morquio A Syndrome)	inborn errors of metabolism or immune disorders	24-Jul-2009	28-Apr-2014				x	x		BioMarin International Limited
Voraxaze	glucarpidase	Adjunctive treatment in patients at risk of methotrexate toxicity	other conditions	3-Feb-2003	11-Jan-2022			x	x	x		Serb
Votubia	everolimus	Treatment of tuberous sclerosis	cancers	4-Aug-2010	2-Sep-2011	x	x (11/2015)		x	x	x (08/2020)	Novartis Europharm Limited
Voxzogo	vosoritide	Treatment of achondroplasia	inborn errors of metabolism or immune disorders	24-Jan-2013	26-Aug-2021				x	x		BioMarin International Limited
Vpriv	velaglucerase alfa	Treatment of Gaucher disease	inborn errors of metabolism or immune disorders	9-Jun-2010	26-Aug-2010				x	x	x (08/2016)	Takeda Pharmaceuticals International AG Ireland Branch
Vyndaqel	tafamidis	Treatment of senile systemic amyloidosis	inborn errors of metabolism or immune disorders	8-Nov-2012	17-Feb-2020			x				Pfizer Europe MA EEIG
Vyxeos liposomal	daunorubicin / cytarabine	Treatment of acute myeloid leukaemia	cancers	11-Jan-2012	23-Aug-2018							Jazz Pharmaceuticals Ireland Ltd
Wakix	pitolisant hydrochloride	Treatment of narcolepsy	inborn errors of metabolism or immune disorders	10-Jul-2007	31-Mar-2016							Bioprojet Pharma

Indication for children (adults are aged 18 years or older)

Tradename	Active substance	Indication	Classification	Designation date	Date of authorisation	Conditional marketing authorisation	Marketing authorisation witch to non-conditional	Authorisation under exceptional circumstances	New orphan medicine	May 2022	2 additional years of market exclusivity as paediatric reward	Marketing authorisation holder
Waylivra	volanesorsen	Treatment of familial chylomicronemia syndrome	inborn errors of metabolism or immune disorders	19-Feb-2014	3-May-2019	x						Akcea Therapeutics Ireland Limited
Xermelo	telotristat	Treatment of carcinoid syndrome	other conditions	8-Oct-2009	18-Sep-2017							Ipsen Pharma
Xospata	gilteritinib	Treatment of acute myeloid leukaemia	cancers	17-Jan-2018	24-Oct-2019							Astellas Pharma Europe B.V.
Yescarta	axicabtagene ciloleucel	Treatment of diffuse large B cell lymphoma	cancers	16-Dec-2014	23-Aug-2018							Kite Pharma EU B.V.
Yescarta	axicabtagene ciloleucel	Treatment of primary mediastinal large B-cell lymphoma	cancers	9-Oct-2015	23-Aug-2018							Kite Pharma EU B.V.
Zejula	niraparib	Treatment of ovarian cancer	cancers	4-Aug-2010	16-Nov-2017							GlaxoSmithKline (Ireland) Limited
Zolgensma	onasemnogene abeparvovec	Treatment of spinal muscular atrophy	inborn errors of metabolism or immune disorders	19-Jun-2015	18-May-2020	x	x (05/2022)		x	x		Novartis Gene Therapies EU Limited

Table S2 Medicinal products with marketing authorisation for orphan medicines with expired orphan designation

									Indication for children (adults are aged 18 years or older)			
**Tradename**	**Active substance**	**Indication**	**Classification**	**Designation date**	**Date of authorisation**	**Conditional marketing authorisation**	**Marketing authorisation switch to non-conditional**	**Authorisation under exceptional circumstances**	**New orphan medicine**	**May 2022**	**2 additional years of market exclusivity as paediatric reward**	**Marketing authorisation holder**
Afinitor	everolimus	Treatment of renal cell carcinoma	cancers	5-Jun-2007	3.8.2009							Novartis Europharm Limited
Aldurazyme	laronidase	Treatment of Mucopolysaccharidosis, type I	inborn errors of metabolism or immune disorders	14-Feb-2001	10.6.2003				x	x		Genzyme Europe B.V.
Arzerra	ofatumumab	Treatment of chronic lymphocytic leukaemia	cancers	7-Nov-2008	19.4.2010	x	x (04/2015)					Novartis Europharm Limited
Atriance	nelarabine	Treatment of acute lymphoblastic leukaemia	cancers	16-Jun-2005	22.8.2007			x	x	x		Novartis Europharm Limited
Bavencio	avelumab	Treatment of Merkel cell carcinoma	cancers	14-Dec-2015	18.9.2017	x	x (08/2020)					Merck Europe B.V.
Bosulif	bosutinib	Treatment of chronic myeloid leukaemia	cancers	4-Aug-2010	27.3.2013	x	x (04/2022)					Pfizer Limited
Bronchitol	mannitol	Treatment of cystic fibrosis	inborn errors of metabolism or immune disorders	7-Nov-2005	13.4.2012							Pharmaxis Europe Limited
Busilvex	busulfan	Busilvex followed by cyclophosphamide (BuCy2) is indicated as conditioning treatment prior to conventional haematopoietic progenitor cell transplantation (HPCT) in adult patients when the combination is considered the best available option. Busilvex followed by cyclophosphamide (BuCy4) or melphalan (BuMel) is indicated as conditioning treatment prior to conventional haematopoietic progenitor cell transplantation in paediatric patients.	other conditions	29-Dec-2000	9.7.2003					x		Pierre Fabre Médicament
Carbaglu	carglumic acid	Treatment of isovaleric acidaemia	inborn errors of metabolism or immune disorders	7-Nov-2008	27.5.2011				x	x		Recordati Rare Diseases
Carbaglu	carglumic acid	Treatment of methylmalonic acidaemia	inborn errors of metabolism or immune disorders	7-Nov-2008	27.5.2011				x	x		Recordati Rare Diseases

									Indication for children (adults are aged 18 years or older)			
**Tradename**	**Active substance**	**Indication**	**Classification**	**Designation date**	**Date of authorisation**	**Conditional marketing authorisation**	**Marketing authorisation switch to non-conditional**	**Authorisation under exceptional circumstances**	**New orphan medicine**	**May 2022**	**2 additional years of market exclusivity as paediatric reward**	**Marketing authorisation holder**
Carbaglu	carglumic acid	Treatment of propionic acidaemia	inborn errors of metabolism or immune disorders	7-Nov-2008	27.5.2011				x	x		Recordati Rare Diseases
Carbaglu	carglumic acid	Treatment of N-acetylglutamate synthetase (NAGS) deficiency	inborn errors of metabolism or immune disorders	18-Oct-2000	24.1.2003				x	x		Orphan Europe S.A.R.L.
Cayston	aztreonam	Treatment of gram negative bacteria lung infections in cystic fibrosis	other conditions	21-Jun-2004	21.9.2009	x	x (09/2011)			x		Gilead Sciences Ireland UC
Ceplene	histamine dihydrochloride	Treatment of acute myeloid leukaemia	cancers	11-Apr-2005	7.10.2008			x				Noventia Pharma Srl
Cyramza	ramucirumab	Treatment of gastric cancer	cancers	4-Jul-2012	19.12.2014							Eli Lilly Nederland B.V.
Cystadane	betaine anhydrous	Treatment of homocystinuria	inborn errors of metabolism or immune disorders	9-Jul-2001	15.2.2007				x	x		Orphan Europe S.A.R.L.
Diacomit	stiripentol	Treatment of severe myoclonic epilepsy in infancy	other conditions	5-Dec-2001	4.1.2007	x	x (01/2014)		x	x		Biocodex
Elaprase	Idursulfase	Treatment of Mucopolysaccharidosis, type II (Hunter Syndrome)	inborn errors of metabolism or immune disorders	11-Dec-2001	8.1.2007			x	x	x		Shire Human Genetic Therapies AB
Esbriet	pirfenidone	Treatment of idiopathic pulmonary fibrosis	other conditions	16-Nov-2004	28.2.2011							Roche Registration GmbH
Evoltra	clofarabine	Treatment of acute lymphoblastic leukaemia	cancers	5-Feb-2002	29.5.2006			x	x	x		Genzyme Europe B.V.
Exjade	deferasirox	Treatment of chronic iron overload requiring chelation therapy	other conditions	13-Mar-2002	28.8.2006				x	x		Novartis Europharm Limited
Fabrazyme	agalsidase beta	Treatment of Fabry disease	inborn errors of metabolism or immune disorders	8-Aug-2000	3.8.2001				x	x		Genzyme Europe B.V.
Firazyr	icatibant	treatment of angioedema	inborn errors of metabolism or immune disorders	17-Feb-2003	11.7.2008					x	x (10/2017)	Shire Pharmaceuticals Ireland Limited
Firdapse	amifampridine	Treatment of Lambert-Eaton myasthenic syndrome	inborn errors of metabolism or immune disorders	18-Nov-2002	23.12.2009			x				BioMarin International Limited
Gliolan	5-aminolevulinic acid hydrochloride	Intra-operative photodynamic diagnosis of residual glioma	other conditions	13-Nov-2002	7.9.2007							medac Gesellschaft für klinische Spezialpräparate mbH

Indication for children (adults are aged 18 years or older)

Tradename	Active substance	Indication	Classification	Designation date	Date of authorisation	Conditional marketing authorisation	Marketing authorisation switch to non-conditional	Authorisation under exceptional circumstances	New orphan medicine	May 2022	2 additional years of market exclusivity as paediatric reward	Marketing authorisation holder
Glivec	imatinib	Treatment of malignant gastrointestinal stromal tumours	cancers	20-Nov-2001	24.5.2002							Novartis Europharm Limited
Glivec	imatinib	Treatment of acute lymphoblastic leukaemia	cancers	26-Aug-2005	13.9.2006					x		Novartis Europharm Limited
Glivec	imatinib	Treatment of dermatofibrosarcoma protuberans	cancers	26-Aug-2005	13.9.2006							Novartis Europharm Limited
Glivec	imatinib	Treatment of chronic eosinophilic leukaemia and the hypereosinophilic syndrome	cancers	28-Oct-2005	28.11.2006							Novartis Europharm Limited
Glivec	imatinib	Treatment of myelodysplastic / myeloproliferative diseases	cancers	23-Dec-2005	28.11.2006							Novartis Europharm Limited
Glivec	imatinib	Treatment of chronic myeloid leukaemia	cancers	14-Feb-2001	7.11.2001					x		Novartis Europharm Limited
Glybera	alipogene tiparvovec	Treatment of lipoprotein lipase deficiency	inborn errors of metabolism or immune disorders	8-Mar-2004	25.10.2012			x				uniQure Biopharma B.V.
Ilaris	canakinumab	Treatment of cryopirin-associated periodic syndromes (Familial Cold Urticaria Syndrome (FCUS), Muckle-Wells Syndrome (MWS), and Neonatal Onset Multisystem Inflammatory Disease (NOMID), also known as Chronic Infantile Neurological Cutaneous	inborn errors of metabolism or immune disorders	20-Mar-2007	23.10.2009			x	x	x		Novartis Europharm Limited
Imbruvica	ibrutinib	Treatment of chronic lymphocytic leukaemia	cancers	26-Apr-2012	21.10.2014							Janssen-Cilag International NV
Imbruvica	ibrutinib	Treatment of mantle cell lymphoma	cancers	12-Mar-2013	21.10.2014							Janssen-Cilag International NV
Imbruvica	ibrutinib	Treatment of lymphoplasmacytic lymphoma	cancers	29-Apr-2014	3.7.2015							Janssen-Cilag International NV
Increlex	mecasermin	Treatment of primary insulin-like growth factor-1 deficiency due to molecular or genetic defects	inborn errors of metabolism or immune disorders	22-May-2006	3.8.2007			x	x	x		Ipsen Pharma
Inovelon	rufinamide	Treatment of Lennox-Gastaut syndrome	other conditions	20-Oct-2004	16.1.2007				x	x	x (01/2017)	Eisai GmbH
Jakavi	ruxolitinib	Treatment of chronic idiopathic myelofibrosis	cancers	7-Nov-2008	23.8.2012							Novartis Europharm Limited

Indication for children (adults are aged 18 years or older)

Tradename	Active substance	Indication	Classification	Designation date	Date of authorisation	Conditional marketing authorisation	Marketing authorisation switch to non-conditional	Authorisation under exceptional circumstances	New orphan medicine	May 2022	2 additional years of market exclusivity as paediatric reward	Marketing authorisation holder
Jakavi	ruxolitinib	Treatment of myelofibrosis secondary to polycythemia vera or essential thrombocythemia	cancers	3-Apr-2009	23.8.2012							Novartis Europharm Limited
Kolbam	cholic acid	Treatment of inborn errors of primary bile acid synthesis responsive to treatment with cholic acid	inborn errors of metabolism or immune disorders	28-Oct-2009	4.4.2014			x	x	x		Retrophin Europe Limited
Kuvan	sapropterin dihydrochloride	Treatment of hyperphenylalaninemia	inborn errors of metabolism or immune disorders	8-Jun-2004	2.12.2008				x	x	x (06/2015)	BioMarin International Limited
Lartruvo	olaratumab	Treatment of soft tissue sarcoma	cancers	12-Feb-2015	9.11.2016	x						Eli Lilly Nederland B.V.
Lenvima	lenvatinib	Treatment of follicular thyroid cancer	cancers	26-Apr-2013	28.5.2015							Eisai GmbH
Lenvima	lenvatinib	Treatment of papillary thyroid cancer	cancers	26-Apr-2013	28.5.2015							Eisai GmbH
Litak	cladribine	Treatment of indolent non-Hodgkin's lymphoma	cancers	18-Sep-2001	14.4.2004				x			Lipomed GmbH
Lumoxiti	moxetumomab pasudotox	Treatment of hairy cell leukaemia	cancers	4-Dec-2008	8.2.2021			x				AstraZeneca AB
Lynparza	olaparib	Treatment of ovarian cancer	cancers	6-Dec-2007	16.12.2014							AstraZeneca AB
Lysodren	mitotane	Treatment of adrenal cortical carcinoma	cancers	12-Jun-2002	28.4.2004							Laboratoire HRA Pharma
Mepact	mifamurtide	Treatment of osteosarcoma	cancers	21-Jun-2004	6.3.2009				x	x		Takeda France SAS
Mozobil	plerixafor	Treatment to mobilize progenitor cells prior to stem cell transplantation	other conditions	20-Oct-2004	31.7.2009					x	x (05/2019)	Genzyme Europe B.V.
Myozyme	alglucosidase alfa	Treatment of Glycogen Storage Disease type II (Pompe's disease)	inborn errors of metabolism or immune disorders	14-Feb-2001	29.3.2006				x	x		Genzyme Europe B.V.
Naglazyme	galsulfase	Treatment of Mucopolysaccharidosis, type VI (Maroteaux-Lamy Syndrome)	inborn errors of metabolism or immune disorders	14-Feb-2001	24.1.2006			x	x	x		BioMarin Europe Ltd
Nexavar	sorafenib	Treatment of renal cell carcinoma	cancers	29-Jul-2004	19.7.2006							Bayer Pharma AG
Nexavar	sorafenib	Treatment of hepatocellular carcinoma	cancers	11-Apr-2006	29.10.2007							Bayer AG
Nplate	romiplostim	Treatment of idiopathic thrombocytopenic purpura	inborn errors of metabolism or immune disorders	27-May-2005	4.2.2009					x		Amgen Europe B.V.
Ofev	nintedanib	Treatment of idiopathic pulmonary fibrosis	other conditions	26-Apr-2013	15.1.2015							Boehringer Ingelheim

Indication for children (adults are aged 18 years or older)

Tradename	Active substance	Indication	Classification	Designation date	Date of authorisation	Conditional marketing authorisation	Marketing authorisation switch to non-conditional	Authorisation under exceptional circumstances	New orphan medicine	May 2022	2 additional years of market exclusivity as paediatric reward	Marketing authorisation holder
												International GmbH
Ofev	nintedanib	Treatment of systemic sclerosis	inborn errors of metabolism or immune disorders	29-Aug-2016	17.4.2020							Boehringer Ingelheim International GmbH
Onsenal	celecoxib	Treatment of Familial Adenomatous Polyposis	cancers	20-Nov-2001	17.10.2003							Pfizer Limited
Orfadin	nitisinone	Treatment of tyrosinaemia type I	inborn errors of metabolism or immune disorders	29-Dec-2000	21.2.2005			x	x	x		Swedish Orphan Biovitrum International AB
Pedea	ibuprofen	Treatment of patent ductus arteriosus	other conditions	14-Feb-2001	29.7.2004				x	x		Orphan Europe S.A.R.L.
Peyona	caffeine citrate	Treatment of primary apnoea of premature newborns	other conditions	17-Feb-2003	2.7.2009				x	x		Chiesi Farmaceutici S.p.A.
PhotoBarr	porfimer sodium	Treatment of high-grade dysplasia in Barrett’s Esophagus	other conditions	6-Mar-2002	25.3.2004							Pinnacle Biologics B.V.
Plenadren	hydrocortisone	Treatment of adrenal insufficiency	inborn errors of metabolism or immune disorders	22-May-2006	3.11.2011							Shire Services BVBA
Prialt	ziconotide	Treatment of chronic pain requiring intraspinal analgesia	other conditions	9-Jul-2001	21.2.2005			x				Eisai Limited
Replagal	agalsidase alfa	Treatment of Fabry disease	inborn errors of metabolism or immune disorders	8-Aug-2000	3.8.2001							Shire Human Genetic Therapies AB
Revatio	sildenafil	Treatment of pulmonary arterial hypertension and chronic thromboembolic pulmonary hypertension	other conditions	12-Dec-2003	28.10.2005			x		x		Pfizer Limited
Revlimid	lenalidomide	Treatment of multiple myeloma	cancers	12-Dec-2003	14.6.2007							Celgene Europe Limited
Revlimid	lenalidomide	Treatment of myelodysplastic syndromes	cancers	8-Mar-2004	13.6.2013							Celgene Europe B.V.
Revlimid	lenalidomide	Treatment of mantle cell lymphoma	cancers	27-Oct-2011	8.7.2016							Celgene Europe B.V.
Revolade	eltrombopag	Treatment of idiopathic thrombocytopenic purpura	inborn errors of metabolism or immune disorders	3-Aug-2007	11.3.2010					x		GlaxoSmithKline Trading Services Limited
Rilonacept Regeneron	rilonacept	Treatment of cryopirin-associated periodic syndromes (Familial Cold Urticaria Syndrome (FCUS), Muckle-Wells Syndrome (MWS), and	inborn errors of metabolism or immune disorders	10-Jul-2007	23.10.2009			x	x	x		Regeneron UK Limited

									Indication for children (adults are aged 18 years or older)			
**Tradename**	**Active substance**	**Indication**	**Classification**	**Designation date**	**Date of authorisation**	**Conditional marketing authorisation**	**Marketing authorisation switch to non-conditional**	**Authorisation under exceptional circumstances**	**New orphan medicine**	**May 2022**	**2 additional years of market exclusivity as paediatric reward**	**Marketing authorisation holder**
		Neonatal Onset Multisystem Inflammatory Disease (NOMID), also known as Chronic Infantile Neurological Cutaneous Articular Syndrome (CINCA)										
Rubraca	rucaparib	Treatment of ovarian cancer	cancers	10-Oct-2012	24.5.2018	x						Clovis Oncology UK Limited
Savene	dexrazoxane	Treatment of anthracycline extravasations	other conditions	19-Sep-2001	28.7.2006							Norgine B.V.
Signifor	pasireotide	Treatment of Cushing's disease	other conditions	8-Oct-2009	24.4.2012							Recordati Rare Diseases
Siklos	hydroxycarbamide	Treatment of sickle cell syndrome	inborn errors of metabolism or immune disorders	9-Jul-2003	29.6.2007				x	x		Addmedica SAS
Skysona	elivaldogene autotemcel	Treatment of adrenoleukodystrophy	inborn errors of metabolism or immune disorders	6-Jun-2012	16.7.2021				x	x		bluebird bio (Netherlands) B.V.
Soliris	eculizumab	Treatment of paroxysmal nocturnal haemoglobinuria	inborn errors of metabolism or immune disorders	17-Oct-2003	20.6.2007					x	x (07/2015)	Alexion Europe SAS
Somavert	pegvisomant	Treatment of acromegaly	other conditions	14-Feb-2001	13.11.2002							Pfizer Limited
Sprycel	dasatinib	Treatment of acute lymphoblastic leukaemia	cancers	23-Dec-2005	20.11.2006					x		Bristol-Myers Squibb Pharma EEIG
Sprycel	dasatinib	Treatment of chronic myeloid leukaemia	cancers	23-Dec-2005	20.11.2006					x		Bristol-Myers Squibb Pharma EEIG
Sutent	sunitinib	Treatment of malignant gastrointestinal stromal tumours	cancers	10-Mar-2005	11.1.2007							Pfizer Limited
Tasigna	nilotinib	Treatment of chronic myeloid leukaemia	cancers	22-May-2006	19.11.2007					x	x (11/2017)	Novartis Europharm Limited
Tepadina	thiotepa	Conditioning treatment prior to haematopoietic progenitor cell transplantation	other conditions	29-Jan-2007	15.3.2010				x	x		ADIENNE S.r.l.
Thalidomide BMS	thalidomide	Treatment of multiple myeloma	cancers	20-Nov-2001	16.4.2008							Celgene Europe Limited
Thelin	sitaxentan sodium	Treatment of pulmonary arterial hypertension and chronic thromboembolic pulmonary hypertension	other conditions	21-Oct-2004	10.8.2006							Pfizer Limited
Torisel	temsirolimus	Treatment of renal cell carcinoma	cancers	6-Apr-2006	19.11.2007							Pfizer Limited

									Indication for children (adults are aged 18 years or older)			
**Tradename**	**Active substance**	**Indication**	**Classification**	**Designation date**	**Date of authorisation**	**Conditional marketing authorisation**	**Marketing authorisation switch to non-conditional**	**Authorisation under exceptional circumstances**	**New orphan medicine**	**May 2022**	**2 additional years of market exclusivity as paediatric reward**	**Marketing authorisation holder**
Torisel	temsirolimus	Treatment of mantle cell lymphoma	cancers	6-Nov-2006	21.8.2009							Pfizer Europe MA EEIG
Tracleer	bosentan	Treatment of pulmonary arterial hypertension and chronic thromboembolic pulmonary hypertension	other conditions	14-Feb-2001	15.5.2002				x	x		Actelion Registration Ltd
Tracleer	bosentan	Treatment of systemic sclerosis	inborn errors of metabolism or immune disorders	17-Mar-2003	7.6.2007							Actelion Registration Ltd
Trisenox	arsenic trioxide	Treatment of acute promyelocytic leukaemia	cancers	18-Oct-2000	5.3.2002							Cephalon Europe
Unituxin	dinutuximab	Treatment of neuroblastoma	cancers	21-Jun-2011	14.8.2015				x	x		United Therapeutics Europe Ltd
Venclyxto	venetoclax	Treatment of chronic lymphocytic leukaemia	cancers	6-Nov-2012	5.12.2016	x	x (11/2018)					AbbVie Deutschland GmbH & Co. KG
Ventavis	iloprost	Treatment of primary and of the following forms of secondary pulmonary hypertension: connective tissue disease pulmonary hypertension, drug-induced pulmonary hypertension, portopulmonary hypertension, pulmonary hypertension associated with congenital heart disease, chronic thromboembolic pulmonary hypertension	other conditions	29-Dec-2000	16.9.2003							Bayer Pharma AG
Vidaza	azacitidine	Treatment of myelodysplastic syndromes	cancers	6-Feb-2002	17.12.2008							Celgene Europe B.V.
Vidaza	azacitidine	Treatment of acute myeloid leukaemia	cancers	29-Nov-2007	17.12.2008							Celgene Europe B.V.
Volibris	ambrisentan	Treatment of pulmonary arterial hypertension and chronic thromboembolic pulmonary hypertension	other conditions	11-Apr-2005	21.4.2008					x		Glaxo Group Ltd
Vyndaqel	tafamidis	Treatment of familial amyloid polyneuropathy	inborn errors of metabolism or immune disorders	28-Aug-2006	16.11.2011			x				Pfizer Specialty UK Ltd
Wilzin	zinc acetate dihydrate	Treatment of Wilson's disease	inborn errors of metabolism or immune disorders	31-Jul-2001	13.10.2004				x	x		Orphan Europe S.A.R.L.

									Indication for children (adults are aged 18 years or older)			
**Tradename**	**Active substance**	**Indication**	**Classification**	**Designation date**	**Date of authorisation**	**Conditional marketing authorisation**	**Marketing authorisation switch to non-conditional**	**Authorisation under exceptional circumstances**	**New orphan medicine**	**May 2022**	**2 additional years of market exclusivity as paediatric reward**	**Marketing authorisation holder**
Xagrid	anagrelide	Treatment of essential thrombocythaemia	inborn errors of metabolism or immune disorders	29-Dec-2000	16.11.2004			x			x (11/2014)	Shire Pharmaceutical Development Limited
Xaluprine	mercaptopurine	Treatment of acute lymphoblastic leukaemia	cancers	30-Apr-2009	9.3.2012				x	x		Nova Laboratories Ireland Limited
Xyrem	sodium oxybate	Treatment of narcolepsy	inborn errors of metabolism or immune disorders	3-Feb-2003	13.10.2005					x		UCB Pharma Ltd
Yondelis	trabectedin	Treatment of soft tissue sarcoma	cancers	30-May-2001	17.9.2007			x				Pharma Mar S.A.
Yondelis	trabectedin	Treatment of ovarian cancer	cancers	17-Oct-2003	28.10.2009			x				Pharma Mar S.A.
Zalmoxis	allogeneic T cells genetically modified with a retroviral vector encoding for a truncated form of the human low affinity nerve growth factor receptor (ΔLNGFR) and the herpes simplex I virus thymidine kinase (HSV-TK Mut2)	Adjunctive treatment in hematopoietic cell transplantation	other conditions	20-Oct-2003	18.8.2016	x						MolMed S.p.A.
Zavesca	miglustat	Treatment of Gaucher Disease	inborn errors of metabolism or immune disorders	18-Oct-2000	20.11.2002							Actelion Registration Ltd
Zavesca	miglustat	Treatment of Niemann-Pick disease, type C	inborn errors of metabolism or immune disorders	16-Feb-2006	26.1.2009				x	x		Janssen-Cilag International NV
Zynteglo	betibeglogene autotemcel	Treatment of beta-thalassaemia intermedia and major	inborn errors of metabolism or immune disorders	24-Jan-2013	29.5.2019	x			x	x		bluebird bio (Netherlands) B.V.
