# Supplementary material for: EMA approved orphan medicines since the implementation of the orphan legislation
Source: Orphanet J Rare Dis. 2025 Jun 2;20:266. doi: 10.1186/s13023-025-03756-7 (PMC12131831; doi:10.1186/s13023-025-03756-7)
Supplement: Supplementary file 2 — Additional file 2: The research material. Table S3. Orphan medicinal products on the market in Finland in May 2024 which had valid orphan designation in May 2022. Table S4. Orphan medicinal products on the market in Finland in May 2024 which had expired orphan designation in May 2022. [file 13023_2025_3756_MOESM2_ESM.pdf]

Supplemental Information: Additional file 2 – The research material

Orphanet Journal of Rare Diseases

EMA approved orphan medicines since the implementation of the orphan legislation, Orphanet Journal of Rare Diseases

Eveliina Hahl [1,*], Terhi Kurko [2], Hanna Koskinen [2], Marja Airaksinen [1] and Kati Sarnola [2]

* Correspondence: eveliina.hahl@helsinki.fi
[1] Faculty of Pharmacy, University of Helsinki, Helsinki, Finland
[2] Research Unit, The Social Insurance Institution of Finland, Helsinki, Finland

Contents
Table S3 Orphan medicinal products on the market in Finland in May 2024 which had valid orphan designation in May 2022
Table S4 Orphan medicinal products on the market in Finland in May 2024 which had expired orphan designation in May 2022

Table S3 Orphan medicinal products on the market in Finland in May 2024 which had valid orphan designation in May 2022

Tradename[1]	Active substance	Reimbursable	Outpatient medicine[2]	Medicine used in inpatient care[2]
Adcetris	brentuximab vedotin	no		x
Adempas	riociguat	yes	x	
Alofisel	darvadstrocel	no	x	
Alprolix	eftrenonacog alfa	yes	x	
Arikayce liposomal	amikacin	no	x	
Aspaveli	pegcetacoplan	yes		x
Besponsa	inotuzumab ozogamicin	no		x
Blincyto	blinatumomab	no		x
Cablivi	caplacizumab	no	x	
Cerdelga	eliglustat	yes	x	
Cometriq	cabozantinib	yes	x	
Cresemba	isavuconazole	yes	x	

Tradename[1]	Active substance	Reimbursable	Outpatient medicine[2]	Medicine used in inpatient care[2]
Crysvita	burosumab	yes	x	
Cystadrops	mercaptamine	no	x	
Dacogen	decitabine	no		x
Darzalex	daratumumab	yes	x	
Defitelio	defibrotide	no		x
Enspryng	satralizumab	no	x	
Epidyolex	cannabidiol	yes	x	
Evrysdi	risdiplam	yes	x	
Fintepla	fenfluramine	yes	x	
Galafold	migalastat	yes	x	
Gazyvaro	obinutuzumab	no		x
Hepcludex	bulevirtide	no	x	
Iclusig	ponatinib	yes	x	
Idefirix	imlifidase	no		x
Imnovid (Pomalidomide Celgene)	pomalidomide	yes	x	
Isturisa	osilodrostat	no	x	
Jorveza	budesonide	yes	x	
Kaftrio	ivacaftor / tezacaftor / elexacaftor	yes	x	
Kalydeco	ivacaftor	yes	x	
Kanuma	sebelipase alfa	no		x
Ketoconazole HRA	ketoconazole	yes	x	
Kimmtrak	tebentafusp	no		x
Koselugo	selumetinib	no	x	
Kymriah	tisagenlecleucel	no		x
Kyprolis	carfilzomib	no		x
Ledaga	chlormethine	yes	x	
Minjuvi	tafasitamab	no		x

Tradename[1]	Active substance	Reimbursable	Outpatient medicine[2]	Medicine used in inpatient care[2]
Mylotarg	gemtuzumab ozogamicin	no		x
Natpar	parathyroid hormone	no	x	
Nexavar	sorafenib	yes	x	
NexoBrid	Concentrate of proteolytic enzymes enriched in bromelain	no	x	
Ngenla	somatrogon	yes	x	
Ninlaro	ixazomib	yes	x	
Ocaliva	obeticholic acid	yes	x	
Onivyde pegylated liposomal	irinotecan	no		x
Opsumit	macitentan	yes	x	
Oxbryta	voxelotor	no	x	
Pemazyre	pemigatinib	yes	x	
Polivy	polatuzumab vedotin	no		x
Poteligeo	mogamulizumab	no		x
Prevymis	letermovir	yes	x	
Procysbi	mercaptamine (cysteamine bitartrate)	no	x	
Qarziba	dinutuximab beta	no		x
Ravicti	glycerol phenylbutyrate	yes	x	
Raxone	idebenone	yes	x	
Reblozyl	luspatercept	no	x	
Revestive	teduglutide	yes	x	
Rydapt	midostaurin	yes	x	
Signifor	pasireotide	yes	x	
Sirturo	bedaquiline	no	x	
Soliris	eculizumab	no		x
Spinraza	nusinersen	no	x	
Strensiq	asfotase alfa	yes	x	

Tradename[1]	Active substance	Reimbursable	Outpatient medicine[2]	Medicine used in inpatient care[2]
Sylvant	siltuximab	no		x
Symkevi	tezacaftor/ivacaftor	yes	x	
Takhzyro	lanadelumab	yes	x	
Tavneos	avacopan	yes	x	
Tecartus	brexucabtagene autoleucel	no		x
Tobi Podhaler	tobramycin	yes	x	
Translarna	ataluren	yes	x	
Trecondi	treosulfan	no		x
Voraxaze	glucarpidase	no	x	
Votubia	everolimus	yes	x	
Vpriv	velaglucerase alfa	yes		x
Vyndaqel	tafamidis	yes	x	
Vyxeos liposomal	daunorubicin / cytarabine	no		x
Wakix	pitolisant hydrochloride	yes	x	
Xermelo	telotristat	no	x	
Xospata	gilteritinib	yes	x	
Yescarta	axicabtagene ciloleucel	no		x
Zejula	niraparib	yes	x	
Zolgensma	onasemnogene abeparvovec	no		x

[1]Every novel orphan medicine is only one time in the table. Not including extensions of indications.

[2]Classified by dosage form. Infusions are classified as medicine used in inpatient care; other medicines are classified as outpatient medicines.

Table S4 Orphan medicinal products on the market in Finland in May 2024 which had expired orphan designation in May 2022

Tradename[1]	Active substance	Reimbursable	Outpatient medicine[2]	Medicine used in inpatient care[2]
Afinitor	everolimus	yes	x	
Aldurazyme	laronidase	no		x
Atriance	nelarabine	no		x
Bavencio	avelumab	no		x
Bosulif	bosutinib	yes	x	
Carbaglu	carglumic acid	no	x	
Cyramza	ramucirumab	no		x
Cystadane	betaine anhydrous	yes	x	
Diacomit	stiripentol	yes	x	
Elaprase	Idursulfase	no		x
Esbriet	pirfenidone	no	x	
Exjade	deferasirox	yes	x	
Fabrazyme	agalsidase beta	yes		x
Firazyr	icatibant	yes	x	
Firdapse	amifampridine	no	x	
Gliolan	5-aminolevulinic acid hydrochloride	no	x	
Glivec	imatinib	yes	x	
Ilaris	canakinumab	no	x	
Imbruvica	ibrutinib	yes	x	
Inovelon	rufinamide	yes	x	
Jakavi	ruxolitinib	yes	x	
Lenvima	lenvatinib	yes	x	
Litak	cladribine	no	x	
Lynparza	olaparib	yes	x	
Lysodren	mitotane	yes	x	
Mozobil	plerixafor	no	x	

Tradename[1]	Active substance	Reimbursable	Outpatient medicine[2]	Medicine used in inpatient care[2]
Myozyme	alglucosidase alfa	no		x
Nexavar	sorafenib	yes	x	
Nplate	romiplostim	yes	x	
Ofev	nintedanib	yes	x	
Orfadin	nitisinone	yes	x	
Pedea	ibuprofen	no	x	
Peyona	caffeine citrate	no	x	
Prialt	ziconotide	no		x
Replagal	agalsidase alfa	yes		x
Revatio	sildenafil	yes	x	
Revlimid	lenalidomide	no	x	
Revolade	eltrombopag	yes	x	
Savene	dexrazoxane	no		x
Signifor	pasireotide	yes	x	
Siklos	hydroxycarbamide	no	x	
Soliris	eculizumab	no		x
Somavert	pegvisomant	yes	x	
Sprycel	dasatinib	no	x	
Sutent	sunitinib	yes	x	
Tasigna	nilotinib	yes	x	
Tepadina	thiotepa	no		x
Thalidomide BMS	thalidomide	no	x	
Torisel	temsirolimus	no		x
Tracleer	bosentan	yes	x	
Trisenox	arsenic trioxide	no		x
Venclyxto	venetoclax	yes	x	
Ventavis	iloprost	yes	x	

Tradename[1]	Active substance	Reimbursable	Outpatient medicine[2]	Medicine used in inpatient care[2]
Vidaza	azacitidine	no	x	
Volibris	ambrisentan	yes	x	
Vyndaqel	tafamidis	yes	x	
Wilzin	zinc acetate dihydrate	no	x	
Xagrid	anagrelide	yes	x	
Xaluprine	mercaptopurine	yes	x	
Xyrem	sodium oxybate	yes	x	
Yondelis	trabectedin	no		x
Zavesca	miglustat	yes	x	

[1]Every novel orphan medicine is only one time in the table. Not including extensions of indications.

[2]Classified by dosage form. Infusions are classified as medicine used in inpatient care; other medicines are classified as outpatient medicines.
